# FEA Framework for Short-Fiber-Reinforced Thermoplastics with Process-Induced Anisotropic Thermomechanical Properties

**DOI:** 10.3390/polym18212768

**Published:** 2026-11-06

**Authors:** Damian Bodniewicz, Mirosław Bocian

**Affiliations:** 1 Department of Mechanics, Materials and Biomedical Engineering, Wrocław University of Science and Technology, Smoluchowskiego 25, 50-372 Wrocław, Poland; damian.bodniewicz@pwr.edu.pl; 2 Centre of Materials Science and Metal Forming, Wrocław University of Science and Technology, Łukasiewicza 5, 50-351 Wrocław, Poland

**Keywords:** short-fiber-reinforced thermoplastics, SFRT, injection molding, fiber orientation tensor, anisotropic material modeling, thermomechanical properties, finite element analysis, PA66-GF30, temperature effects, moisture effects

## Abstract

This study presents a finite element framework for injection-molded short-fiber-reinforced thermoplastics that accounts for process-induced anisotropy, temperature, and moisture. A fully oriented material model was combined with fiber orientation tensors from injection-molding simulations to determine local orthotropic thermomechanical properties of PA66-GF30. The model employs micromechanical formulations for elasticity, strength, and thermal expansion. Matrix Young’s modulus, Poisson’s ratio, tensile strength, and coefficient of linear thermal expansion were identified simultaneously through inverse calibration using standardized composite data. Before calibration, prediction accuracy depended strongly on property, temperature, and moisture condition, with the largest deviations occurring at 120 °C and for conditioned-state tensile strength. After calibration, the directly targeted longitudinal Young’s modulus and tensile strength agreed with their targets within ±0.4%. The non-targeted transverse modulus and tensile strength agreed with auxiliary estimates within ±5.4% and ±1.9%, respectively. For the dry-as-molded state, the directional thermal expansion coefficients agreed with their calibration targets within ±5.2%. For the conditioned state, dry-as-molded thermal expansion values were adopted as targets, and deviations remained within ±5.9%. These results demonstrate successful calibration and internal consistency, while independent validation requires directional measurements not used during identification. The framework enables practical mapping of environment-dependent anisotropic properties in structural finite element analysis.

## 1. Introduction

Thermoplastic polymers are gaining increasing importance in engineering applications due to their favorable mechanical and thermal properties, high manufacturing efficiency, and potential to replace traditional materials in selected components. Their mechanical performance can be significantly improved by introducing reinforcing fillers, particularly chopped fibers such as glass, carbon, or aramid fibers. Short-fiber-reinforced thermoplastics (SFRTs) are usually produced by injection molding, which leads to local anisotropy in thermomechanical properties. During injection molding, the initially disordered and entangled polymer chains in the plasticized matrix may become partially oriented along the local flow field, particularly in regions subjected to high shear rates and rapid cooling. At the same time, the reinforcing fibers interact dynamically with the flowing polymer matrix and develop a process-dependent orientation state governed by the local flow history [1,2,3,4]. As a result, variations in fiber orientation and, to a lesser extent, molecular orientation of the polymer matrix can be expected across the thickness of the part walls. These manufacturing-induced microstructural features may contribute to residual stresses and post-molding deformation, which can subsequently undergo partial relaxation due to the viscoelastic nature of thermoplastics, especially at elevated temperatures or under sustained loading [4,5,6,7].

Considering the through-thickness variations in material structure and the resulting local anisotropy induced by the cavity filling process, treating an injection-molded part as an isotropic continuum may lead to significant errors in strength assessment, even when empirical correction factors are employed [8,9]. In industrial practice, commonly used finite element analysis (FEA) workflows neglect manufacturing-induced local material characteristics or represent them only approximately, which may lead to insufficient accuracy in predicting the initial state and strength response of injection-molded structures.

The mechanical properties of thermoplastics are highly sensitive to external factors such as temperature and deformation rate. This behavior is strongly related to the thermally activated mobility of polymer chains and becomes particularly important in the vicinity of the glass transition temperature (*T*_g_) [10]. *T*_g_ represents a transition range in which the amorphous phase of a polymer changes from a glassy, energy-elastic state with strongly restricted segmental mobility to a more compliant, entropy-elastic state characterized by increased molecular rearrangement capability [11]. Below *T*_g_, thermoplastics generally exhibit higher stiffness and strength, while their deformation is governed mainly by limited chain rearrangement and bond stretching. As the temperature approaches and exceeds *T*_g_, relative segmental motions become increasingly active, which results in a significant reduction in stiffness and strength, as well as an increased contribution of viscoelastic effects such as creep and stress relaxation [7]. Consequently, these materials can fail in both brittle and ductile manners, depending on the molecular state and the ability of polymer chains to rearrange under load. Accordingly, the assumption of a single, constant allowable load for a polymer structure under varying thermal conditions may lead to non-conservative or inaccurate assessments, especially when the service temperature range approaches the *T*_g_ of the polymer matrix. In summary, evaluating complex stress states in such components requires an appropriate strength criterion that accounts for condition-dependent brittle or ductile material behavior and the local anisotropic strength resulting from the direction and degree of fiber orientation.

A specific subgroup of thermoplastics is the family of polyamides. These widely used materials for technical components exhibit valuable mechanical and thermal properties, which fluctuate depending on the moisture content within the molded part. In polyamides, absorbed moisture acts as a plasticizer, increasing the mobility of polymer chains and shifting *T*_g_ toward lower values [11]. As a result, the mechanical response of the material may change significantly even at the same exposure temperature, depending on whether the polymer is in the dry-as-molded, conditioned, or saturated state. As the moisture content increases, polyamides generally show reduced stiffness and tensile strength, while increased ductility can be observed [11,12]. Thus, the analysis of deformation and stress states in polyamide-based components should account for at least two material conditions: the dry-as-molded state and the conditioned state, or, in extreme cases, the saturated state. This is because components made from water-absorbing materials gradually absorb moisture from the environment during service or climatic chamber conditioning, which leads to fluctuations in their mechanical properties over time.

In addition to temperature- and moisture-related changes in material behavior, the numerical modeling of components made from SFRTs must also account for manufacturing-induced local features and defects. One of the most important examples is the weld line. A weld line is formed where separate polymer melt fronts meet, for example after flow separation caused by an obstacle, multiple injection points, or changes in cross-sectional geometry along the flow path [2,13]. The presence of weld lines usually leads to a local decrease in stiffness and strength. This reduction results from surface notches, fiber orientations unfavorable relative to the loading direction, and weakened bonding between the converging melt fronts. The latter effect is associated with factors such as cooling of the joining fronts, accumulation of contaminants and/or trapped air between them, or unfavorable injection process parameters [13,14].

In engineering practice, internal stresses in injection-molded thermoplastic parts are often associated primarily with external mechanical or thermal loads. However, residual stresses remaining after the manufacturing process may also contribute significantly to the total stress state [5]. The main sources of residual stresses include nonuniform cooling, packing pressure, flow-induced effects near solidifying walls, and interactions between the polymer matrix and the reinforcing fibers [6]. Consequently, the as-molded component may exhibit both post-molding deformation and a residual stress field. This may result in dimensional deviations from product specifications and assembly problems. Additionally, the presence of high tensile stresses on the component’s surface can lead to stress cracking shortly after mold opening, or constitute a significant, although difficult-to-quantify, contribution to the operational stress state. In mechanically fastened polymer structures, additional local stresses may also be introduced during assembly, for example during thread forming by self-tapping screws, and these stresses may interact with the time-dependent response of the polymer material [15].

The phenomena discussed above indicate that engineering approaches developed for isotropic structural materials should not be directly transferred to injection-molded SFRTs [16]. Nevertheless, commonly used strength assessment procedures for polymer components often rely on simplified isotropic material descriptions and neglect the local anisotropy induced by fiber orientation. In addition, post-production effects such as residual stresses and warpage, as well as injection-molding defects including weld lines, local density variations, sink marks, and voids, are frequently omitted during the design phase. Although these simplifications reduce computational cost and model complexity, they may lead to significant discrepancies between simulation and experimental results.

Similar challenges arise in the fatigue analysis of thermoplastic components subjected to cyclic loading. Depending on the loading frequency and amplitude, self-heating and subsequent material softening may occur [17], while commonly used mean stress correction methods may be inappropriate for this class of materials [18]. Therefore, fatigue assessment also requires a local material description that accounts for anisotropy, environmental sensitivity, and the local post-production state.

In summary, simplified industrial FEA approaches may introduce significant inaccuracies in static and fatigue strength predictions, which can affect both component design decisions and the feasibility of metal-to-polymer substitution. To address these limitations, the authors propose a methodology for the local static and fatigue strength assessment of injection-molded SFRT structures. The methodology is based on the determination of spatially varying anisotropic thermomechanical properties as a function of the local fiber orientation state.

Fiber orientation can be described in its most general form by a probability density function defined over the unit sphere [3]. However, such a representation is computationally impractical for routine numerical analyses. Consequently, injection-molding simulations typically employ second-order fiber orientation tensors (FOTs), which provide a compact statistical description of the local degree and directionality of reinforcement [3,19]. In the proposed framework, these tensors are used together with the thermomechanical properties of a fully oriented material model (FOMM), which represents the idealized case of complete fiber alignment along a single direction. The effective local material properties are then estimated by orientation averaging of the FOMM properties using the local second-order FOT as the primary descriptor of the fiber orientation state. Where fourth-order orientation information is required for the transformation or homogenization of tensorial material quantities, the fourth-order fiber orientation tensor is reconstructed from the second-order FOT using the Orthotropic Smooth Closure (OSC) approximation [19]. The OSC approximation is used consistently throughout the primary modeling, calibration, and property-mapping procedures presented in this study. To assess the robustness of this choice, the principal calculations were additionally repeated using the Orthotropic Fitted Closure (OFC) approximation, without modifying or recalibrating the remaining model inputs. Finally, the presented procedure enables spatially varying anisotropic thermomechanical properties to be generated in a form suitable for structural FEA [20].

Commercial material databases generally provide isotropic properties or effective longitudinal properties measured on standardized injection-molded specimens. Such data are insufficient to define the complete local orthotropic material description required for structural FEA. An isotropic linear-elastic model requires two independent elastic constants, whereas a general three-dimensional orthotropic model requires nine independent engineering constants. Even under the transversely isotropic assumption adopted for the FOMM, five independent elastic constants are required. Moreover, the longitudinal properties measured on injection-molded specimens represent the effective response of a process-dependent and spatially non-uniform fiber orientation state rather than the properties of an ideally aligned material. Consequently, a single isotropic or effective longitudinal database value cannot be uniquely transformed into the complete local orthotropic property set without additional micromechanical assumptions, directional measurements, or inverse identification combined with a known representative FOT.

This article focuses on the development of an integrated material-modeling framework for FOT-based orientation averaging and homogenization. The framework builds upon established micromechanical formulations rather than introducing a new micromechanical model. Specifically, it describes the preparation of the FOMM, the use of FOTs obtained from injection-molding simulations, and the incorporation of temperature- and moisture-dependent thermomechanical properties. The contribution of the study lies in integrating these elements with inverse calibration based on composite-level reference data within a unified workflow that generates local orthotropic material properties for structural FEA. Furthermore, the paper demonstrates how selected physical effects resulting from local variations in fiber orientation can be captured in detailed FEA of reinforced thermoplastic components.

While the overall methodology is intended to support static and fatigue strength assessment, the present paper is restricted to the determination and numerical implementation of local anisotropic thermomechanical properties for short-term quasi-static analysis. The structural FEA demonstrations therefore employ an orthotropic linear-elastic material description and do not account for time-dependent viscoelastic or viscoplastic deformation, creep, or stress relaxation. The inclusion of these effects would require additional time-, temperature-, moisture-, and orientation-dependent material data and a dedicated constitutive formulation, which are beyond the scope of the present study. Consequently, the current framework should not be used to assess the long-term response of components subjected to sustained loads. Future work will extend the methodology toward time-dependent material behavior, local multidirectional strength assessment, fatigue, and reduction factors associated with injection-molding defects.

## 2. Materials and Methods

As briefly discussed in the Introduction, the proposed simulation framework for SFRT structures consists of material model preparation, injection-molding simulation, transfer of fiber orientation data, structural FEA, and subsequent local strength assessment. The present paper focuses primarily on the preparation and numerical implementation of the anisotropic thermomechanical material model. Two alternative FOMM preparation routes are considered: a non-calibrated route based on initial constituent properties and micromechanical models, and a calibration-based route employing inverse identification from composite-level reference data. The final local static and fatigue strength assessment is considered part of the target methodology and will be addressed in future work.

In the present work, the FOMM is defined as an idealized transversely isotropic material state in which all reinforcing fibers are aligned with the local *x*_1_ direction. In this fully aligned state, the second-order fiber orientation tensor is defined by *a*_11_ = 1, *a*_22_ = 0, and *a*_33_ = 0, with all off-diagonal components equal to zero. For the directly injection-molded dog-bone specimen, *x*_1_ is aligned with the longitudinal specimen axis and nominal mold-filling direction, *x*_2_ denotes the transverse in-plane direction, and *x*_3_ denotes the through-thickness direction. The quantities *a*_11_, *a*_22_, and *a*_33_ are the diagonal FOT components resolved along these axes.

These coordinate-dependent components are distinguished from the ordered principal FOT values *λ*_1_ ≥ *λ*_2_ ≥ *λ*_3_, whose associated eigenvectors define the corresponding principal directions. In general, *a_ii_* ≠ *λ_i_*, unless the selected coordinate axes coincide with the local principal directions of the FOT. Such coincidence occurs in the idealized fully aligned FOMM state but is not assumed for a general local orientation state.

The local second-order FOT, obtained from injection-molding analysis, is subsequently used as the basis for FOT-based orientation averaging of the FOMM properties, following the closure-based homogenization procedure described above [3,19,20]. The complete target workflow is presented in Figure 1.

Residual stresses and other process-induced defects are included as elements of the target methodology but are not transferred to or evaluated in the structural FEA demonstrations presented in this study.

The proposed approach (excluding the injection-molding analysis and the structural FEA solution itself) has been implemented using an in-house program written in APDL (ANSYS Parametric Design Language). The program enables the transfer and use of process-induced fields and local process data in structural FEA, including fiber orientation, residual stresses, moisture distribution, local defects, and relevant temperature and pressure histories. These data can be used to assign element-specific material properties and initial conditions. The program also supports the post-processing of FEA results, including local strength and fatigue assessment based on multidirectional failure criteria.

In the numerical demonstrations presented in this study, only fiber-orientation-induced anisotropy was mapped and evaluated. A spatially uniform moisture state was assumed, while residual-stress transfer and the other process-related data shown in Figure 1 were not activated. Non-uniform moisture fields can nevertheless be incorporated, provided that the local moisture distribution and moisture-dependent material data are available. It should be noted that adequate through-thickness discretization is required to resolve the resulting moisture and orientation gradients. When residual stresses are transferred, their possible relaxation before operational loading should be considered using an appropriate material-specific, time- and temperature-dependent model. However, the exported stress definition must be verified, as relaxation may already have been considered in the injection-molding simulation, to avoid accounting for it twice.

### 2.1. Preparation of the Fully Oriented Material Model (FOMM)

This study aimed to determine the anisotropic thermomechanical properties of the theoretical FOMM representing PA66 reinforced with 30 wt.% glass fibers (PA66-GF30), as functions of exposure temperature and moisture content. This is particularly important for polyamide-based materials, for which changes in moisture content may modify chain mobility and shift the *T*_g_, thereby affecting stiffness, strength, and deformation capability. It should be noted that the required test data (particularly those dependent on the direction of applied mechanical or thermal loads) are typically not officially provided by material suppliers. However, they are essential for conducting numerical stiffness and strength analyses of reinforced thermoplastic components, taking into account the local material anisotropy resulting from fiber orientation and its degree after the injection molding process. Such data can be obtained through dedicated in-house testing or from commercial material databases and simulation packages. However, these sources do not always provide the complete directional property set required for local anisotropic FEA. An alternative approach is to theoretically predict these properties using suitable micromechanical models.

The second challenge associated with the use of experimental data to determine the FOMM lies in the practical impossibility of obtaining specimens exhibiting idealized FOT components, such as *a*_11_ = 1, *a*_22_ = 0, *a*_33_ = 0. In realistic injection-molded parts, the fiber orientation is inherently heterogeneous and varies across both the thickness and the in-plane dimensions of the specimen. This spatial variation results from local processing conditions during manufacturing, including gradients in viscosity, temperature, pressure, and shear rate. As a result, the measured mechanical response reflects the effective behavior of a complex, non-uniform orientation state rather than that of a purely aligned microstructure. This makes it difficult to directly derive material properties corresponding to a fully oriented configuration based solely on standard experimental tests, such as tensile testing. This issue is examined later in this study by analyzing variations in the FOT components associated with different specimen preparation routes. In particular, specimens of comparable geometry produced using different injection-molding strategies are compared to evaluate the influence of manufacturing conditions on fiber orientation.

In summary, even when experimental data from mechanical testing are available, it remains necessary to estimate the properties corresponding to a theoretical fully oriented state. Such estimations are essential for the development of predictive material models and for understanding the bounds of mechanical performance.

In the present study, the orthotropic properties of the FOMM were determined using selected micromechanical models. These approaches treat reinforced thermoplastics as composite structures. Consequently, the thermomechanical properties of the material can be predicted based on the properties of the polymer matrix and the reinforcing fibers, while additionally accounting for the geometry and volume fraction of the reinforcement. The following micromechanical models were used to determine the required FOMM properties:

- orthotropic elastic properties: Halpin–Tsai model [21];
- orthotropic strength: Van Hattum–Bernardo model [22];
- orthotropic thermal expansion: Rosen–Hashin model [23].

The investigated material contains 30 wt.% glass fibers. Because the applied micromechanical models require the reinforcement content to be expressed as a fiber volume fraction, the manufacturer-specified mass fraction was converted according to

(1) $$V_{f}=\frac{w_{f}/\rho_{f}}{w_{f}/\rho_{f}+\left(1-w_{f}\right)/\rho_{m}},$$

where *V_f_* is the glass-fiber volume fraction, *w_f_* is the glass-fiber mass fraction, and *ρ_f_* and *ρ_m_* are the densities of the glass fibers and PA66 matrix, respectively. The values *w_f_* = 0.30, $\rho_{f}=2.54\;\frac{g}{{\mathrm{cm}}^{3}}$, and $\rho_{m}=1.15\;\frac{g}{{\mathrm{cm}}^{3}}$ were used, resulting in *V_f_* = 0.163. The resulting fiber volume fraction was used in all micromechanical calculations.

An effective fiber aspect ratio of *a_r_* = *L_f_*/*d_f_* = 25 was used as a fixed input in the stiffness and strength models for all investigated conditions. Since the fiber dimensions were not measured directly, this value represents an effective modeling parameter.

To determine the composite properties using the selected models, the required constituent properties must be specified for both the PA66 matrix and the reinforcing fibers. Table 1 summarizes the properties of the non-calibrated FOMM obtained directly from the initial constituent properties and the selected micromechanical models.

The *x*_1_ axis defines the fiber-alignment direction of the idealized fully oriented state. In the condensed notation used for the engineering constants, properties with the subscript 11 correspond to loading or thermal expansion along *x*_1_. The *x*_2_ axis lies in the specimen plane and is transverse to *x*_1_, whereas *x*_3_ denotes the through-thickness direction. Owing to the assumed transverse isotropy of the FOMM about the *x*_1_ axis, the properties in the *x*_2_ and *x*_3_ directions are assumed to be identical. Because anisotropic engineering constants may exceed isotropic ranges, the resulting orthotropic stiffness and compliance matrices were checked for thermodynamic admissibility before FEA implementation.

### 2.2. Reference Material Data Used for FOMM Calibration and Evaluation

The proposed approach is founded on the use of input material data for FEA from sources commonly used in industrial practice, such as the CAMPUS Plastics database [24]. CAMPUS is a widely used material information system for the plastics industry, in which supplier-provided data are generated according to uniform international testing standards to ensure comparability across materials and producers. For many thermoplastic materials, a comprehensive set of stiffness and strength data is available over a wide temperature range, as well as for both dry-as-molded and conditioned states in the case of moisture-sensitive materials. However, directional data required for a complete description of anisotropic material behavior are not provided. The tensile stress–strain curves and longitudinal stiffness and strength data available in such databases are commonly obtained from standardized injection-molded tensile specimens. Such specimens may exhibit preferential fiber orientation along the specimen axis and therefore may not represent the general directional response of an injection-molded component. As a consequence, these specimens exhibit a high degree of fiber orientation, resulting in elevated stiffness and strength in the loading direction, although these values remain lower than those corresponding to a fully oriented material state. Hence, the available data may be misleading for engineers, as they represent a near upper-bound estimate of material stiffness and strength rather than the general structural performance of the material. In contrast, mechanical properties in directions perpendicular to the main fiber orientation, as well as those in weld-line regions, are not captured in commonly used material databases, which therefore do not allow for a reliable assessment of the weakest regions of the component.

Temperature-dependent stiffness (Figure 2) and ultimate tensile strength (Figure 3) of PA66-GF30 were derived from CAMPUS Plastics stress–strain data [24].

Based on the available temperature-dependent mechanical data, an apparent mechanical transition temperature (*T*_a_) associated with the glass-transition-related softening of the PA66 matrix was estimated to be approximately 45 °C in the dry-as-molded state, with the corresponding transition range extending from 34.4 °C to 61.9 °C. In the conditioned state, the *T*_a_ was reduced to approximately 5 °C, with a transition range from −5.1 °C to 19.2 °C. This shift is consistent with the plasticizing effect of absorbed moisture on the PA66 matrix and provides a reference for interpreting the temperature-dependent mechanical response. These values should not be interpreted as directly measured calorimetric (DSC) or dynamic-mechanical (DMA) *T*_g_ values, but rather as effective transition indicators derived from the temperature dependence of the mechanical response.

In addition, the mechanical properties corresponding to the transverse in-plane direction, *x*_2_, perpendicular to the longitudinal specimen axis and nominal flow direction *x*_1_, were approximated using the auxiliary estimation procedure described in Section 2.2.1. The resulting values were included in Figure 2 and Figure 3 as model-assisted auxiliary transverse-property estimates and were not used as calibration targets.

From the perspective of structural FEA, one of the key thermal properties required is the material’s coefficient of linear thermal expansion (α). Therefore, this parameter must be properly accounted for and estimated when experimental data are unavailable. For the investigated PA66-GF30 material, directional values of α can be obtained from the CAMPUS Plastics database, including values along the primary fiber orientation and in the transverse direction (according to ISO 11359-1 and ISO 11359-2 [25,26]). However, the available data are limited to the dry-as-molded state (Figure 4).

A concise summary of the collected reference thermomechanical data for PA66-GF30 at three selected temperature levels is presented in Table 2. The table includes stiffness and strength data for both dry-as-molded and conditioned states, whereas independently measured directional coefficients of linear thermal expansion are available only for the dry-as-molded state. This dataset was used to calibrate and evaluate the proposed FOMM-based approach after FOT-based orientation averaging. The directly available longitudinal mechanical properties were used as calibration targets for both moisture conditions. The transverse mechanical properties obtained using the auxiliary estimation procedure were used only to evaluate the corresponding non-targeted model predictions. For the dry-as-molded state, the measured values of $\alpha_{11}^{\mathrm{ref}}$ and $\alpha_{22}^{\mathrm{ref}}$ were used as direct calibration targets. In the absence of corresponding conditioned-state data, the dry-as-molded values at the same temperature were adopted as assumed calibration targets for the conditioned state. They should therefore not be interpreted as independently measured conditioned-state properties. The effects of exposure temperature, moisture condition, and proximity to the estimated *T_a_* of the reinforced PA66 are discussed in the following subsections.

It should be emphasized that the data summarized in Table 2 should not be interpreted as FOMM properties. They represent specimen-level reference values obtained from standardized tests and, for the transverse mechanical properties, from an auxiliary estimation procedure. In contrast, the values listed in Table 1 correspond to the theoretical non-calibrated FOMM properties obtained directly from the initial constituent properties and the selected micromechanical models. Therefore, evaluation of model accuracy requires FOT-based orientation averaging using a representative orientation state of the tested specimen, as discussed in Section 3.

#### 2.2.1. Auxiliary Estimation of Transverse Mechanical Properties

Direct experimental values of the effective transverse Young’s modulus and ultimate tensile strength were unavailable for the directly injection-molded PA66-GF30 specimens considered in this study. Experimental determination of these reference values is challenging because transverse specimens are typically machined from injection-molded plates. Since their fiber orientation differs from that of directly injection-molded tensile specimens, the measured properties cannot be transferred directly between the two configurations.

The transverse reference values were therefore approximated using an auxiliary procedure developed prior to the calibration performed in the present study. The procedure was established using proprietary quasi-static tensile data for several short-glass-fiber-reinforced thermoplastics. The material systems and test conditions represented in the original dataset are summarized in Table 3. PA66-GF30 was not included as a separate material system in this dataset.

All experimental specimens were machined in selected directions from injection-molded plates and tested under quasi-static tensile loading according to ISO 527-1 and ISO 527-2 [27,28] or a corresponding laboratory implementation. Commercial material grades, detailed processing parameters, complete fiber orientation distributions, and individual experimental results cannot be disclosed because of confidentiality restrictions.

The properties measured on the machined specimens reflected their process-induced fiber orientation and could therefore not be assigned directly to the longitudinal and transverse specimen configurations considered in Figure 2 and Figure 3. Injection-molding simulations of the corresponding plates were performed to determine representative FOT for the specimen locations and cutting directions used in the experiments. The experimental responses and the corresponding simulated tensors were then used to identify FOMM, which served as an intermediate material description.

The estimated FOMM was subsequently combined, without further parameter adjustment, with the separately simulated FOT of the directly injection-molded longitudinal and transverse specimens. This transfer provided the effective *E*_11_ and *E*_22_ values corresponding to the specimen configurations presented in Figure 2 and the effective *UTS*_11_ and *UTS*_22_ values corresponding to those presented in Figure 3.

Based on the results obtained for the materials and test conditions listed in Table 3, the relative reductions in effective transverse stiffness and tensile strength were correlated with the glass-fiber mass fraction *w_f_*, the material-specific apparent mechanical transition temperature *T_a_*, and the test temperature *T*:

(2) *D_E_* = *g_E_*(*w_f_*,*T_a_*,*T*),

(3) *D_UTS_* = *g_UTS_*(*w_f_*,*T_a_*,*T*).

Due to confidentiality restrictions associated with the underlying industrial dataset, the fitted coefficients and individual data points used to determine *g_E_* and *g_UTS_* cannot be disclosed. Consequently, Equations (2) and (3) are not proposed as generally reproducible predictive models. Within the present study, they are used exclusively to generate internal engineering consistency references for the transverse response of the investigated PA66-GF30 specimen.

For the PA66-GF30 investigated in the present study, the auxiliary transverse reference values were calculated from the directly available longitudinal reference properties as

(4) $$E_{22}^{\mathrm{ref},\mathrm{aux}}=\left[1-D_{E}(w_{f},T_{a},T)\right]E_{11}^{\mathrm{ref}},$$

(5) $$\mathrm{UT}S_{22}^{\mathrm{ref},\mathrm{aux}}=\left[1-D_{\mathrm{UTS}}(w_{f},T_{a},T)\right]\mathrm{UT}S_{11}^{\mathrm{ref}}.$$

The resulting $E_{22}^{\mathrm{ref},\mathrm{aux}}$ and $\mathrm{UT}S_{22}^{\mathrm{ref},\mathrm{aux}}$ values represent auxiliary estimates of the effective transverse response of the considered directly injection-molded specimen configurations. They are neither direct transverse measurements of the PA66-GF30 investigated in this study nor directional properties of the FOMM.

The empirical scope of the auxiliary procedure is limited to short-glass-fiber-reinforced thermoplastics, nominal reinforcement contents of approximately 30–50 wt.%, the temperature and moisture conditions represented in Table 3, and quasi-static tensile loading. Its application is therefore more justified for interpolation within this domain than for extrapolation to substantially different fiber contents, other reinforcement types, continuous-fiber composites, strain-rate-dependent or impact loading, or environmental conditions outside the original dataset.

The accuracy of the auxiliary estimates depends primarily on the simulated fiber orientation states and the adopted micromechanical and orientation-averaging assumptions. The procedure has not been independently validated against direct transverse tensile measurements for the specific PA66-GF30 grade investigated in the present study. The resulting values should therefore be regarded as auxiliary consistency references rather than independently validated experimental reference data.

#### 2.2.2. Effect of Exposure Temperature on Mechanical Properties

The reference stiffness and strength data show a pronounced decrease with increasing exposure temperature. This trend is consistent with the temperature-dependent molecular mobility of the PA66 matrix, discussed previously in relation to the glass-transition-related softening behavior. As the exposure temperature increases and approaches or exceeds the transition range of the amorphous phase, segmental motions become progressively more active. In the dry-as-molded state, the estimated *T*_a_ of approximately 45 °C, with a transition range from 34.4 °C to 61.9 °C, indicates that the investigated temperature range extends from a predominantly glassy mechanical response at −20 °C to a temperature well above the transition region at 120 °C. This reduces intermolecular constraints in the polymer matrix and lowers the efficiency of stress transfer between the matrix and the reinforcing fibers. As a result, both Young’s modulus and ultimate tensile strength decrease with increasing temperature.

For the investigated PA66-GF30, this effect is clearly visible in the collected reference data. In the dry-as-molded state, increasing the exposure temperature from −20 °C to 120 °C resulted in a reduction in the nominal longitudinal Young’s modulus $E_{11}^{\mathrm{ref}}$ by approximately 58%, while the corresponding ultimate tensile strength ${\mathrm{UTS}}_{11}^{\mathrm{ref}}$ decreased by approximately 56%. The estimated transverse stiffness showed an even stronger temperature sensitivity: over the same temperature range, $E_{22}^{\mathrm{ref},\mathrm{aux}}$ decreased by approximately 77%. In contrast, the estimated transverse ultimate tensile strength ${\mathrm{UTS}}_{22}^{\mathrm{ref},\mathrm{aux}}$ decreased by approximately 52%, which is comparable to the reduction observed in the loading direction. These results indicate that elevated temperature affects both the matrix-dominated transverse response and the fiber-oriented loading direction. However, the longitudinal stiffness remains partially stabilized by the reinforcing phase, whereas the transverse response is governed more directly by the temperature-dependent behavior of the polymer matrix.

A comparable temperature-dependent reduction was also observed in the conditioned state. In this case, the estimated *T*_a_ was approximately 5 °C, with a transition range from −5.1 °C to 19.2 °C. Consequently, the material at 20 °C is already close to or slightly above the transition region, which explains the pronounced reduction in stiffness and strength relative to the low-temperature state. Relative to the value at −20 °C, the nominal longitudinal Young’s modulus $E_{11}^{\mathrm{ref}}$ decreased by approximately 34% at 20 °C and by approximately 65.5% at 120 °C. The corresponding reduction in ${\mathrm{UTS}}_{11}^{\mathrm{ref}}$ was approximately 37% at 20 °C and 65% at 120 °C. The estimated transverse Young’s modulus $E_{22}^{\mathrm{ref},\mathrm{aux}}$ decreased by approximately 55% at 20 °C and by approximately 81% at 120 °C, while ${\mathrm{UTS}}_{22}^{\mathrm{ref},\mathrm{aux}}$ decreased by approximately 35% and 63%, respectively. These results demonstrate that the mechanical response of PA66-GF30 cannot be represented by a single temperature-independent material dataset.

The observed behavior is particularly important from the perspective of FEA. If a single stiffness or strength value is assigned to the material independently of exposure temperature, the resulting model may provide an inaccurate representation of the component response. Depending on the selected reference temperature, this simplification may lead to either an overestimation or underestimation of stiffness, stress redistribution, and load-bearing capacity. This is especially critical in regions with unfavorable local fiber orientations, such as transverse-flow areas or weld-line regions, where the mechanical response is more strongly influenced by the softened polymer matrix or adverse local injection-molding conditions. Therefore, the temperature-dependent material data summarized in Table 2 should be treated as separate input states for the proposed anisotropic FEA methodology rather than as a single averaged material description.

#### 2.2.3. Effect of Moisture Conditioning on Mechanical Properties

In addition to temperature-related effects, moisture absorption generally reduces the mechanical performance of PA66-GF30, particularly at room and elevated temperatures. Analysis of stiffness (Figure 2) and strength (Figure 3) shows that conditioning, corresponding to equilibrium moisture content at room temperature and 50% relative humidity, leads to a clear decrease in both stiffness and strength compared with the dry-as-molded state at 20 °C and 120 °C. At −20 °C, however, the differences between the dry-as-molded and conditioned states are small and may be considered negligible for the present analysis.

This behavior should be interpreted mainly in relation to the moisture-induced shift in the glass-transition-related softening range of the PA66 matrix, represented in this work by the estimated apparent mechanical transition temperature *T*_a_. In the dry-as-molded state, the *T*_a_ is approximately 45 °C, whereas in the conditioned state it decreases to approximately 5 °C. Therefore, at 20 °C the dry-as-molded material remains below the transition range, while the conditioned material is close to or slightly above this range.

At 20 °C, conditioning results in an approximately 30% reduction in both the longitudinal Young’s modulus and the ultimate tensile strength. The transverse, matrix-dominated stiffness is even more sensitive, decreasing by more than 50%, while the transverse strength is reduced by approximately 28%. These results indicate that the moisture-induced reduction in *T*_a_ primarily affects the matrix-dominated response, whereas the longitudinal direction remains partially stabilized by the reinforcing glass fibers.

At 120 °C, both dry-as-molded and conditioned material states are well above their respective estimated *T*_a_ values. Consequently, the additional effect of moisture is partly masked by the already significant thermal softening of the polymer matrix. Nevertheless, at 120 °C conditioning reduces the longitudinal and transverse stiffness by approximately 13.7% and 14.3%, respectively. The corresponding reductions in longitudinal and transverse tensile strength are approximately 22.0% and 23.4%. In contrast, at −20 °C, both material states are below their respective *T*_a_ values; therefore, the observed differences are relatively small and should not be overinterpreted.

The reduction in mechanical properties is primarily attributed to moisture-induced plasticization of the PA66 matrix. Water molecules diffuse into the amorphous regions of the polymer and interact with polar functional groups, particularly amide bonds, weakening hydrogen bonding and other intermolecular interactions that restrict molecular mobility in the dry-as-molded state. As a result, absorbed water increases the free volume, facilitates segmental motion, and lowers the glass-transition range of the PA66 matrix, which is reflected in this work by the reduced estimated *T*_a_ and contributes to the observed decrease in stiffness and strength [11,12,29].

Increased molecular mobility may also promote time-dependent deformation mechanisms such as creep and stress relaxation. Although PA66 is a semicrystalline polymer and its crystalline regions are less directly affected by moisture absorption than the amorphous phase, the softening of the amorphous regions plays a major role in the macroscopic reduction in mechanical performance [12].

Overall, moisture absorption significantly modifies the load-transfer mechanisms in PA66-GF30 by increasing molecular mobility and shifting the glass-transition-related softening range toward lower temperatures, as reflected by the reduced estimated *T*_a_. The reference data show that this effect is most critical at room temperature, where the conditioned material is close to or slightly above its *T*_a_, while the dry-as-molded material remains below it. Therefore, dry-as-molded and conditioned states should be treated as separate material conditions in the numerical analysis of polyamide-based SFRT.

#### 2.2.4. Effect of Exposure Temperature on Thermal Expansion

The reference coefficients of linear thermal expansion (α) for PA66-GF30 in the dry-as-molded state, presented in Figure 4, exhibit a pronounced anisotropic response. The coefficient along the preferred fiber-orientation direction, $\alpha_{11}^{\mathrm{ref}}$, remains low and nearly constant, decreasing only slightly from approximately 28 × 10^−6^ to 24 × 10^−6^ K^−1^, whereas the transverse coefficient ($\alpha_{22}^{\mathrm{ref}}$) is significantly higher and increases strongly with temperature (from about 75 × 10^−6^ to 122 × 10^−6^ K^−1^). This behavior is characteristic of SFRTs and results from the anisotropic microstructure governed by fiber orientation and differences in the thermoelastic properties of the constituent phases. In the main flow/fiber-preferred direction, the thermal expansion response $\alpha_{11}^{\mathrm{ref}}$ is strongly constrained by the glass fibers, which exhibit low intrinsic thermal expansion and high stiffness. Due to their preferential alignment induced during injection molding, the fibers effectively constrain the thermal expansion of the PA66 matrix by carrying thermally induced stresses. Consequently, the overall $\alpha_{11}^{\mathrm{ref}}$ is significantly reduced and only weakly dependent on temperature. This effect is well described by classical micromechanical models of fiber-reinforced composites [21,23].

In contrast, the transverse response ($\alpha_{22}^{\mathrm{ref}}$) is primarily governed by the polymer matrix, as the reinforcing efficiency of the fibers is limited in directions perpendicular to their orientation. The matrix exhibits higher thermal expansion and strong temperature sensitivity due to increasing chain mobility. The rising $\alpha_{22}^{\mathrm{ref}}$ observed in Figure 4 may be further influenced by the following mechanisms [6,29,30]:

- forced lateral expansion, where the volumetric change is redirected perpendicular to the fiber orientation;
- an increase in free volume and enhanced molecular mobility;
- the progressive relaxation of internal constraints and residual stresses generated during cooling, packing, and due to the mismatch in thermal expansion and shrinkage between the fiber and matrix phases.

Overall, the observed anisotropy of α is a direct consequence of the fiber orientation state and constituent interactions, and it must be explicitly considered in thermomechanical modeling of fiber-reinforced polymers.

### 2.3. Inverse Identification of Matrix Properties for FOMM Calibration

The FOMM calibration was formulated as an inverse-identification problem in which selected matrix properties were determined from composite-level reference data. Rather than applying correction factors directly to individual FOMM properties, the complete FOMM property set was recalculated for each trial matrix-property vector using the adopted micromechanical models.

The identification was performed separately for each temperature *T* and moisture condition *h*. The glass-fiber properties, fiber volume fraction, and fiber aspect ratio were kept constant. The optimization vector was defined as

(6) $$p_{T,h}=\left[\begin{array}{c} E_{m,T,h}\\ \nu_{m,T,h}\\ \mathrm{UT}S_{m,T,h}\\ \alpha_{m,T,h} \end{array}\right],\;h\in \{\mathrm{dry},\mathrm{cond}\},$$

where *E_m_*, *ν_m_*, *UTS_m_*, and *α_m_* denote the matrix Young’s modulus, Poisson’s ratio, ultimate tensile strength, and coefficient of linear thermal expansion, respectively. All four parameters were varied simultaneously within each temperature- and moisture-specific optimization problem.

For every trial parameter vector, the orthotropic elastic, strength, and thermal expansion properties of the FOMM were recalculated as

(7) $$C_{T,h}^{\mathrm{FOMM}}=M_{\mathrm{HT}}\left(p_{T,h},q_{f},q_{g}\right),$$

(8) $$U_{T,h}^{\mathrm{FOMM}}=M_{\mathrm{VHB}}\left(p_{T,h},q_{f},q_{g}\right),$$

(9) $$\alpha_{T,h}^{\mathrm{FOMM}}=M_{\mathrm{RH}}\left(p_{T,h},q_{f},q_{g}\right)\;h\in \{\mathrm{dry},\mathrm{cond}\},$$

where ℳ*_HT_*, ℳ*_VHB_*, and ℳ*_RH_* denote the Halpin–Tsai, Van Hattum–Bernardo, and Rosen–Hashin micromechanical operators, respectively. The vectors **q***_f_* and **q***_g_* contain the fixed glass-fiber properties and microstructural parameters, including the fiber volume fraction and fiber aspect ratio. The complete equations of the micromechanical models are provided in the cited references and are not repeated here.

The recalculated FOMM properties were converted into effective specimen-level properties through FOT-based orientation averaging:

(10) $${\hat{X}}_{T,h}^{\mathrm{eff}}=H_{\mathrm{mech}}\left(C_{T,h}^{\mathrm{FOMM}},U_{T,h}^{\mathrm{FOMM}},A^{\mathrm{rep}}\;\right),$$

(11) $${\hat{\alpha }}_{T,h}^{\mathrm{eff}}=H_{\mathrm{th}}\left(\alpha_{T,h}^{\mathrm{FOMM}},A^{\mathrm{rep}}\right),\;h\in \{\mathrm{dry},\mathrm{cond}\}.$$

where ℋ*_mech_* and ℋ*_th_* denote the mechanical and thermal orientation-averaging operators. The same representative FOT of the directly injection-molded specimen, **A***^rep^*, was used in the orientation averaging of all mechanical and thermal properties. Its volume-averaged diagonal components in the specimen coordinate system were *a*_11_ = 0.866, *a*_22_ = 0.069, and *a*_33_ = 0.065, while the off-diagonal components were negligible.

The fourth-order FOT required by the orientation-averaging formulations was reconstructed from the second-order FOT using the Orthotropic Smooth Closure (OSC) approximation [19]. OSC was applied consistently in the inverse identification, specimen-level property evaluation, and component-level property mapping.

A supplementary sensitivity assessment was performed using the Orthotropic Fitted Closure (OFC) approximation [19]. For this assessment, the OSC-calibrated FOMM datasets, second-order FOTs, material conditions, micromechanical formulations, and orientation-averaging procedures were retained without modification. No recalibration was performed using OFC. Consequently, the reported OSC–OFC differences isolate the effect of the closure approximation.

For a predicted property *k*, the relative prediction error was defined as

(12) $$r_{k,T,h}=\frac{{\hat{X}}_{k,T,h}^{\mathrm{eff}}-X_{k,T,h}^{\mathrm{ref}}}{X_{k,T,h}^{\mathrm{ref}}}.$$

where positive and negative values indicate overprediction and underprediction, respectively.

Because directional thermal expansion data were unavailable for the conditioned state, the corresponding dry-as-molded values at the same temperature were adopted as assumed conditioned-state targets:

(13) $$\alpha_{11,T,\mathrm{cond}}^{\mathrm{ref}}=\alpha_{11,T,\mathrm{dry}}^{\mathrm{ref}},$$

(14) $$\alpha_{22,T,\mathrm{cond}}^{\mathrm{ref}}=\alpha_{22,T,\mathrm{dry}}^{\mathrm{ref}}.$$

These assumed composite-level values were used as calibration targets, while *α_m_*_,_*_T_*_,_*_cond_* was identified independently within each conditioned-state optimization.

The objective function included equally weighted absolute relative errors in the effective longitudinal Young’s modulus, longitudinal ultimate tensile strength, and longitudinal and transverse coefficients of linear thermal expansion:

(15) $$J_{T,h}\left(p_{T,h}\right)=|r_{E_{11},T,h}|+|r_{\mathrm{UT}S_{11},T,h}|+|r_{\alpha_{11},T,h}|+|r_{\alpha_{22},T,h}|,\;h\in \{\mathrm{dry},\mathrm{cond}\}.$$

The identified parameter vector was obtained as

(16) $$p_{T,h}^{*}=\arg\min_{p_{T,h}\in \Omega_{T,h}}J_{T,h}\left(p_{T,h}\right),\;h\in \{\mathrm{dry},\mathrm{cond}\},$$

where Ω*_T_*_,_*_h_* denotes the admissible parameter domain. The identified quantities should be interpreted as effective model parameters that minimize the adopted objective function within this domain; uniqueness of the intrinsic constituent properties is not claimed.

The effective transverse Young’s modulus and tensile strength were not included in the objective function. They were evaluated after calibration as non-targeted consistency checks against the auxiliary estimates described in Section 2.2.1.

### 2.4. Minimum Data Requirements for Industrial Application

The following summary is intended primarily for engineers and analysts interested in applying the proposed workflow in an industrial setting, where complete constituent-level and anisotropic material datasets are frequently unavailable. Depending on data availability, FOMM preparation can follow either a non-calibrated constituent-based route or a calibration-based inverse-identification route. These two routes differ mainly in the type of data used to define the matrix response and in the role of the fiber orientation tensor obtained for the reference specimen.

In the non-calibrated route, the FOMM is calculated directly from the independently specified properties of the polymer matrix and fibers, together with the fiber volume fraction and representative fiber geometry. This route can therefore be used without composite-level calibration data, provided that sufficiently reliable constituent properties are available for the considered temperature and moisture condition. A representative FOT of a reference specimen is not required to construct the FOMM itself. However, a local FOT field is required when the resulting fully oriented properties are mapped to an injection-molded component.

In the calibration-based route, selected matrix properties are identified by matching the orientation-averaged model response to the available composite-level reference data. Consequently, this route can be used when the environmental dependence of the neat-matrix properties is incomplete or insufficiently documented. In addition to the reference composite properties, the calibration requires a representative FOT for the specimen from which those properties were obtained. This tensor is necessary to distinguish the influence of constituent properties from the influence of the process-induced fiber orientation. The calibrated quantities should therefore be regarded as effective model parameters rather than as independently measured properties of the neat polymer.

Table 4 summarizes the minimum input data required for both routes. It distinguishes between the data needed to prepare the FOMM, the data needed to perform the calibration, and the local process data needed for component-level structural analysis.

The representative specimen tensor **A***^rep^*, used during calibration, must not be confused with the spatially varying local component tensor field **A**(**x**), used for component-level property mapping.

## 3. Results

A comparison between the theoretical thermomechanical properties of the FOMM, summarized in Table 1, and the corresponding reference data presented in Table 2 reveals noticeable discrepancies. These differences arise from the characteristic fiber orientation states associated with the respective datasets. The former corresponds to an idealized fully oriented material state in which all fibers are aligned with the local *x*_1_ axis. In this coordinate system, the second-order fiber orientation tensor is defined by *a*_11_ = 1, *a*_22_ = 0, and *a*_33_ = 0, with all off-diagonal components equal to zero. This state represents an idealized upper-bound orientation state for properties evaluated along the fiber-alignment direction, rather than a realistic orientation state produced by injection molding.

In contrast, the reference data represent the effective response of a realistic, process-dependent fiber orientation state, which is not fully aligned and strongly depends on the specimen manufacturing process. The primary fiber orientation develops mainly in near-wall regions, where the molten polymer experiences high shear rates associated with velocity and temperature gradients during cavity filling. As a result, a non-uniform and only partially oriented reinforcement structure develops across the specimen thickness, depending significantly on its geometry, the location of the injection gate(s), and the injection molding process parameters.

A higher fraction of material formed near the cavity walls leads to a larger portion of the local cross-section having a high degree of fiber orientation. This explains why reference data obtained from specimens of identical geometry (e.g., according to ISO 20753 [31] or ISO 3167 [32]), but manufactured using different processes (e.g., directly injection-molded specimens or samples machined from an injection-molded plate), may exhibit significantly different thermomechanical properties. Therefore, precise knowledge of the specimen manufacturing method is essential when interpreting reference material data. To demonstrate the influence of specimen preparation on the resulting orientation state, the local FOT distributions obtained for two representative manufacturing routes are compared in the following subsection.

### 3.1. Evaluation of the Local Fiber Orientation Tensor (FOT)

To evaluate the effect of specimen preparation on the predicted fiber orientation state, injection-molding simulations were performed for two cases: a directly injection-molded specimen with a single-sided gate (Figure 5a) and an injection-molded plate (Figure 5b), from which specimens of comparable geometry can be machined from the central region. The through-thickness distributions of the FOT components extracted at the center of the specimen gauge section are compared in Figure 6.

The through-thickness FOT distributions differed substantially between the two manufacturing routes. In the directly injection-molded specimen, the longitudinal component *a*_11_ remained dominant throughout the entire thickness and varied within a relatively narrow range of approximately 0.85–0.92. This result indicates a consistently strong preferential fiber orientation along the longitudinal specimen axis and nominal mold-filling direction *x*_1_. The transverse in-plane component *a*_22_ and the through-thickness component *a*_33_ generally remained below 0.11. A local decrease in *a*_11_ occurred near the specimen mid-plane and was accompanied primarily by an increase in *a*_33_. The distributions were approximately symmetric with respect to the specimen mid-plane.

A substantially different through-thickness structure was observed for the injection-molded plate. In the regions closer to both plate surfaces, *a*_11_ reached approximately 0.83–0.84, whereas *a*_22_ remained at approximately 0.13–0.15. These regions were therefore characterized by preferential fiber orientation along the nominal mold-filling direction *x*_1_. Toward the plate mid-plane, however, *a*_11_ decreased sharply to approximately 0.20, while *a*_22_ increased to approximately 0.76. Consequently, the dominant diagonal FOT component changed from *a*_11_, corresponding to the nominal mold-filling direction *x*_1_, in the outer regions to *a*_22_, corresponding to the transverse in-plane direction *x*_2_, in the core. The through-thickness component *a*_33_ remained small over the entire plate thickness, reaching only approximately 0.04 near the mid-plane.

Thus, the plate exhibits a pronounced layered orientation structure rather than simply a less-oriented core. The relation *a*_11_ > *a*_22_ > *a*_33_ applies to the outer regions, whereas *a*_22_ > *a*_11_ > *a*_33_ is observed near the mid-plane. By contrast, *a*_11_ remains dominant throughout the directly injection-molded specimen.

In both configurations, the highest *a*_11_ values occurred beneath the outer surfaces. This effect was particularly pronounced in the plate, where two approximately symmetric subsurface regions with high *a*_11_ surrounded the *a*_22_-dominated core. The observed layered structure is consistent with through-thickness variations in the local flow conditions during cavity filling. However, Figure 6 alone does not allow the individual contributions of shear flow, fountain flow, cooling, and fiber-interaction effects to be separated.

When the local FOT field is transferred to the structural FEA mesh, the tensor is represented for each finite element by its principal values and the corresponding principal directions. FOT is volume-averaged within each structural finite element during the mapping procedure. Consequently, adequate through-thickness mesh resolution is required to preserve the layered surface–core orientation and stiffness distribution. An insufficient number of elements through the thickness may smooth this gradient and affect stiffness and stress predictions, particularly in thin-walled or bending-dominated structures. Figure 7 presents the mapped principal FOT values *λ*_1_, *λ*_2_, and *λ*_3_, ordered such that *λ*_1_ ≥ *λ*_2_ ≥ *λ*_3_. These values characterize the magnitudes of fiber orientation along the local principal directions and should not be identified directly with the coordinate-dependent components *a*_11_, *a*_22_, and *a*_33_ presented in Figure 6. In regions where a principal direction is closely aligned with one of the specimen coordinate axes, the corresponding principal value may approach the dominant diagonal tensor component. However, the principal values alone do not identify whether the dominant orientation is aligned with *x*_1_, *x*_2_, or *x*_3_; therefore, the change from *x*_1_ in the outer regions of the plate to *x*_2_ near its mid-plane should be interpreted using the coordinate-system-based components shown in Figure 6 or the corresponding principal directions.

For a quantitative specimen-scale comparison, the diagonal tensor components *a*_11_, *a*_22_, and *a*_33_, expressed in the specimen-based coordinate system, were volume-averaged over the finite elements within the gauge section. The resulting values are summarized in Table 5 and visualized in Figure 8 using a common color scale, with the maximum value of 0.866 represented by red.

The volume-averaged values confirm the stronger longitudinal fiber alignment in the directly injection-molded specimen and the greater contribution of transverse in-plane orientation in the specimen machined from the plate. However, they do not reproduce the local reversal between *a*_11_ and *a*_22_ near the plate mid-plane and should therefore be interpreted together with the through-thickness distributions shown in Figure 6b.

### 3.2. Orientation Averaging of Thermomechanical Properties Using the FOT

The theoretical FOMM properties in Table 1 correspond to complete fiber alignment, whereas the reference properties in Table 2 represent the effective response of a process-dependent, partially oriented specimen. Direct comparison of these datasets therefore does not quantify model accuracy. The FOMM properties were first subjected to orientation averaging using the representative FOT of the directly injection-molded specimen.

The effective elastic, strength, and thermal expansion properties were calculated using the orientation-averaging formulations described in [3], [22], and [4], respectively. The predictions were compared with the longitudinal reference data and, for the transverse mechanical response, with the auxiliary estimates obtained using the procedure described in Section 2.2.1. Because the latter were not independent measurements and were not used as calibration targets, they provide non-targeted internal consistency checks rather than independent validation.

#### 3.2.1. Prediction Errors for Thermomechanical Properties

The non-calibrated FOMM properties presented in Table 1 were combined with the representative FOT of the directly injection-molded specimen. The volume-averaged orientation state was defined by *a*_11_ = 0.866, *a*_22_ = 0.069, and *a*_33_ = 0.065, as summarized in Table 5.

The same representative FOT was used for both the mechanical and thermal calculations. The exact manufacturing route and orientation state of the specimens used for the reference thermal expansion measurements could not be established from the available database documentation. Its application to the thermal expansion data therefore constitutes a modeling assumption.

In this study, numerically predicted thermomechanical properties differing from the specified reference values by no more than 10% were considered sufficiently accurate for use in the intended structural FEA workflow. This threshold was applied to elastic moduli, tensile strengths, and coefficients of linear thermal expansion as a common engineering accuracy target for the generated FEA input data. A comparable error level has been reported as acceptable for selected practical FEA applications [33,34]. However, equal relative deviations in elastic properties, strength, and thermal expansion do not necessarily have an equivalent influence on the final structural response. Moreover, the adopted threshold is intended for preliminary and intermediate-level analyses when a complete experimentally characterized anisotropic material dataset is unavailable. It should not be interpreted as a universal validation criterion, safety factor, design allowable, or certification requirement. The required accuracy for a specific application should be established according to the intended model use, the sensitivity of the structural response to the considered material parameter, and the uncertainty associated with the reference data and numerical procedure [35].

The signed relative deviations were calculated as

(17) $$\varepsilon_{X_{\mathrm{ij}}}=\frac{{\hat{X}}_{\mathrm{ij}}^{\mathrm{eff}}-X_{\mathrm{ij}}^{\mathrm{ref}}}{X_{\mathrm{ij}}^{\mathrm{ref}}}*100\%,$$

and, for the transverse mechanical properties evaluated against the auxiliary estimates, as

(18) $$\varepsilon_{X_{22}}^{\mathrm{aux}}=\frac{{\hat{X}}_{22}^{\mathrm{eff}}-X_{22}^{\mathrm{ref},\mathrm{aux}}}{X_{22}^{\mathrm{ref},\mathrm{aux}}}*100\%.$$

where positive and negative values indicate overprediction and underprediction, respectively. The resulting deviations are summarized in Table 6.

The non-calibrated longitudinal Young’s modulus agreed with the reference data at −20 °C and 20 °C, with deviations from −4.3% to −3.1%. At 120 °C, it was underpredicted by 22.7% in the dry-as-molded state and 23.6% in the conditioned state. The transverse modulus exhibited less uniform agreement, with deviations ranging from −31.0% to 0.5%. The largest modulus errors therefore occurred at 120 °C, indicating reduced accuracy of the initial constituent-based elastic description under strongly thermally softened conditions.

The tensile-strength predictions showed a different error pattern. For the dry-as-molded material, the longitudinal and transverse strengths satisfied the adopted ±10% criterion at 20 °C and 120 °C, but not at −20 °C. In the conditioned state, both strength components were overpredicted at all temperatures. The maximum deviations were 47.4% for the longitudinal response and 51.4% for the transverse response, both at 20 °C. This condition lies close to or above the apparent mechanical transition range of the moisture-plasticized matrix, suggesting that the initial constituent properties and the adopted strength model did not fully reproduce the corresponding reduction in matrix-dominated strength. These discrepancies should be considered in light of the limitations of the Van Hattum–Bernardo model, which does not explicitly represent fiber fracture, fiber–matrix debonding, and matrix yielding as separate, progressively evolving damage mechanisms. Fiber fracture is incorporated indirectly through the fiber tensile strength, fiber-length distribution, and critical fiber length in the Kelly–Tyson-based formulation. Matrix strength governs the transverse response and stress-transfer capability under the assumed strong-interface condition; therefore, interfacial debonding is not modeled independently. The resulting unidirectional strengths are combined with the Tsai–Wu failure criterion and orientation averaging to predict the macroscopic composite strength. Moreover, the formulation is essentially temperature-independent and does not account for viscoelasticity, creep, strain-rate effects, or temperature-dependent constituent and interface behavior. Consequently, brittle failure at −20 °C and matrix softening or time-dependent deformation at 120 °C fall outside its original scope and may contribute to the observed deviations.

For the dry-as-molded material, both thermal expansion coefficients satisfied the adopted ±10% criterion at −20 °C and 20 °C. At 120 °C, the deviations increased to 84.6% for ${\hat{\alpha }}_{11}^{\mathrm{eff}}$ and 18.2% for ${\hat{\alpha }}_{22}^{\mathrm{eff}}$. In the conditioned state, the deviations from the assumed targets reached 192.8% for the longitudinal coefficient at 120 °C, whereas the transverse deviations ranged from 11.2% to 15.6%.

The conditioned-state thermal expansion deviations quantify agreement with surrogate targets adopted from the dry-as-molded dataset rather than with independently measured conditioned-state properties. Similarly, the transverse mechanical comparisons depend on auxiliary estimates and should be interpreted as consistency checks.

Overall, the non-calibrated formulation reproduced selected properties with acceptable engineering accuracy, but its performance was not uniform across properties, temperatures, and moisture conditions. The largest discrepancies occurred for stiffness and thermal expansion at 120 °C and for conditioned-state tensile strength. These results motivated the inverse-identification procedure evaluated in the following section.

#### 3.2.2. Calibration of the FOMM by Inverse Identification of Matrix Properties

The inverse-identification procedure described in Section 2.3 was applied independently to each temperature and moisture condition. The resulting calibrated FOMM properties are summarized in Table 7, and the deviations of the corresponding effective properties after FOT-based orientation averaging are presented in Table 8.

The calibrated response closely matched the directly targeted longitudinal mechanical properties. The deviations of ${\hat{E}}_{11}^{\mathrm{eff}}$ and ${\hat{\mathrm{UTS}}}_{11}^{\mathrm{eff}}$ remained within ±0.4% for all investigated temperatures and moisture conditions. Because both properties were included in the objective function, this agreement quantifies calibration fit rather than independent predictive accuracy.

The improvement relative to the non-calibrated formulation was most pronounced for the conditioned-state tensile strength. Before calibration, the longitudinal strength deviations ranged from 25.4% to 47.4%; after calibration, they did not exceed 0.4%.

The non-targeted transverse mechanical properties also showed consistent agreement with the auxiliary estimates. The deviations ranged from −5.4% to 5.3% for ${\hat{E}}_{22}^{\mathrm{eff}}$ and from −1.9% to 1.9% for ${\hat{\mathrm{UTS}}}_{22}^{\mathrm{eff}}$. Both properties therefore remained within the adopted ±10% engineering range under all investigated conditions. Because they were not included in the objective function, these results constitute non-targeted consistency checks. They do not provide independent validation because the transverse reference values were obtained using an auxiliary estimation procedure rather than measured directly.

For the dry-as-molded state, the deviations of ${\hat{\alpha }}_{11}^{\mathrm{eff}}$ were −5.1%, −1.2%, and 5.2% at −20 °C, 20 °C, and 120 °C, respectively. The corresponding deviations of ${\hat{\alpha }}_{22}^{\mathrm{eff}}$ were 5.1%, 1.2%, and −5.2%. Both directional coefficients therefore remained within ±5.2% of their calibration targets.

At each temperature, the residual longitudinal and transverse thermal expansion deviations were approximately equal in magnitude and opposite in sign. This behavior results from representing both directional responses through a single matrix coefficient in the Rosen–Hashin model. The identified *α_m_* therefore represents a compromise that minimizes their combined contribution to the objective function rather than reproducing both targets exactly.

The largest improvement in the dry-as-molded thermal expansion response occurred at 120 °C. The non-calibrated deviations of 84.6% and 18.2% for the longitudinal and transverse coefficients, respectively, were reduced to 5.2% in both directions after calibration.

For the conditioned state, the deviations from the assumed thermal expansion targets were −2.3%, −2.0%, and 5.9% for ${\hat{\alpha }}_{11}^{\mathrm{eff}}$, and 2.3%, 2.0%, and −5.9% for ${\hat{\alpha }}_{22}^{\mathrm{eff}}$. Although the conditioned-state matrix coefficient was identified independently at each temperature, the composite-level targets were adopted from the dry-as-molded data. Consequently, these results quantify agreement with the adopted equal-expansion assumption rather than with independently measured conditioned-state thermal expansion.

Overall, the calibration provided a close fit to the targeted properties and consistent non-targeted transverse predictions. Independent validation of the full anisotropic response nevertheless requires directional measurements not used during identification.

The identified matrix properties should be interpreted as effective parameters of the complete micromechanical and FOT-based orientation-averaging framework. They may compensate for uncertainty in the initial constituent properties, reference data, representative FOT, and adopted micromechanical formulations. They should therefore not be interpreted as independently measured intrinsic properties of unreinforced PA66 or transferred to other micromechanical models without verification.

Despite these limitations, the procedure provides a practical route for preparing temperature- and moisture-dependent FOMM datasets when complete constituent-level properties are unavailable. It retains the micromechanical structure of the material description by identifying underlying matrix parameters rather than applying independent correction factors to the resulting orthotropic properties.

#### 3.2.3. Sensitivity of the Predicted Properties to the Closure Approximation

To assess the sensitivity of the predicted properties to the closure approximation, the effective thermomechanical properties were recalculated using the Orthotropic Fitted Closure (OFC), while retaining the OSC-calibrated FOMM datasets, second-order FOTs, environmental conditions, and orientation-averaging formulations. No inverse re-identification or FOMM recalibration was performed using OFC. The comparison isolates the effect of replacing OSC with OFC in the reconstruction of the fourth-order FOT.

Both OSC and OFC reconstruct the fourth-order FOT in the principal coordinate system of the second-order tensor and preserve its principal directions [19]. However, they use different relations to determine the independent principal components of the fourth-order tensor. OSC applies interpolation between representative uniaxial, planar-random, and spatially random orientation states, whereas OFC employs polynomial relations fitted to fourth-order tensors obtained from numerical solutions of the fiber-orientation probability distribution function.

The comparison was conducted for the two representative specimen orientation states summarized in Table 5. For the directly injection-molded specimen, the volume-averaged second-order FOT components in the gauge section were *a*_11_ = 0.866, *a*_22_ = 0.069, and *a*_33_ = 0.065. For the specimen machined from the injection-molded plate, the corresponding components were *a*_11_ = 0.707, *a*_22_ = 0.267, and *a*_33_ = 0.026. The volume-averaged off-diagonal components were negligible for both specimen configurations. The same second-order tensors were used in the OSC and OFC calculations.

For each specimen configuration, closure-dependent effective properties were calculated for the longitudinal and transverse Young’s moduli, *E*_11_ and *E*_22_; longitudinal and transverse ultimate tensile strengths, *UTS*_11_ and *UTS*_22_; and longitudinal and transverse coefficients of linear thermal expansion, *α*_11_ and *α*_22_. The same orientation-averaging formulations as those employed in the primary analysis were retained: the tensor-based orientation-averaging formulation described in [3] for the elastic properties, the strength formulation described in [22], and the thermal-expansion formulation described in [4]. Calculations were performed at −20 °C, 20 °C, and 120 °C for both the dry-as-molded and conditioned material states.

Because neither closure result was treated as an exact reference solution, their difference was quantified using the symmetric absolute percentage difference:

(19) $$\Delta_{X}^{\mathrm{cl}}=\frac{2|X^{\mathrm{OSC}}-X^{\mathrm{OFC}}|}{|X^{\mathrm{OSC}}|+|X^{\mathrm{OFC}}|}\cdot 100\%,$$

where *X^OSC^* is the effective property obtained using the OSC approximation adopted in the primary analysis, and *X^OFC^* is the corresponding property recalculated using OFC. The parameter $\Delta_{X}^{\mathrm{cl}}$ represents the magnitude of the closure-related difference and does not imply that either closure approximation constitutes an exact reference solution.

The resulting differences for the dry-as-molded and conditioned states are summarized in Table 9 and Table 10, respectively. In the dry-as-molded state, the closure-related differences ranged from 0.4% to 13.0%. The elastic moduli exhibited the greatest sensitivity. For *E*_11_, the differences ranged from 5.6% to 10.0%, whereas for *E*_22_ they ranged from 3.3% to 13.0%. The maximum difference was obtained for the transverse Young’s modulus of the specimen machined from the plate at 120 °C.

The predicted strength properties were less sensitive to the closure approximation. The differences in *UTS*_11_ ranged from 2.5% to 3.4%, while those in *UTS*_22_ ranged from 1.5% to 5.3%. The coefficients of linear thermal expansion exhibited the lowest overall sensitivity. The differences ranged from 0.6% to 3.1% for *α*_11_ and from 0.4% to 3.8% for *α*_22_.

A comparable pattern was observed for the conditioned state. The closure-related differences ranged from 0.4% to 13.7%. The highest sensitivity was again obtained for the elastic response. The differences in *E*_11_ ranged from 5.7% to 10.4%, whereas those in *E*_22_ ranged from 3.3% to 13.7%. The maximum value was obtained for *E*_22_ of the specimen machined from the plate at 120 °C.

For the conditioned-state strength properties, the differences ranged from 2.5% to 3.4% for *UTS*_11_ and from 1.5% to 5.5% for *UTS*_22_. The corresponding differences in thermal expansion ranged from 0.6% to 3.4% for *α*_11_ and from 0.4% to 3.9% for *α*_22_.

The specimen machined from the plate generally exhibited greater closure sensitivity than the directly injection-molded specimen. Based on all six properties and all three temperatures, the average closure-related difference increased from approximately 3.0% for the directly injection-molded specimen to approximately 4.8% for the specimen machined from the plate in the dry-as-molded state. In the conditioned state, the corresponding average differences were approximately 3.4% and 5.2%, respectively. This trend can be associated with the less strongly longitudinal and more distributed orientation state of the specimen machined from the plate. Compared with the directly injection-molded specimen, the machined specimen was characterized by a lower *a*_11_ component and a substantially higher *a*_22_ component. Under this more multiaxial orientation state, differences in reconstruction of the fourth-order orientation tensor have a greater effect on the orientation-averaged properties.

The closure sensitivity also tended to increase at elevated temperature, particularly for the elastic moduli. This result should not be interpreted as temperature dependence of the closure approximation itself, because the same second-order FOT was used at all investigated temperatures. Instead, the influence of the closure formulation on the effective properties depends on the temperature-dependent contrast between the longitudinal and transverse FOMM properties.

Overall, replacement of OSC by OFC did not alter the principal qualitative conclusions of the study. Both closure approximations preserved the relative effects of specimen manufacturing route, material direction, exposure temperature, and moisture condition. The predicted strength properties and thermal expansion coefficients showed comparatively low sensitivity to the closure choice. The elastic moduli were more sensitive, particularly the transverse modulus of the specimen machined from the plate at 120 °C, for which the closure-related difference reached 13.0% in the dry-as-molded state and 13.7% in the conditioned state.

These results support the use of OSC in the primary analysis and demonstrate that the principal conclusions are robust with respect to the selected closure approximation. However, the observed differences in the elastic moduli indicate that closure-related uncertainty should be considered when quantitative stiffness predictions are required for less strongly aligned orientation states, especially at elevated temperature.

### 3.3. Mapping of Anisotropic Properties onto the FEA Model

The calibrated FOMM, prepared according to the proposed workflow presented in Figure 1, was used in the final structural FEA stage to demonstrate the capabilities of the developed mapping software. For this purpose, the two specimen configurations were considered: the directly injection-molded specimen with a single-sided gate (Figure 5a) and the specimen virtually extracted from an injection-molded plate (Figure 5b).

This part of the study had two major goals. The first was to obtain the FOT distribution from the injection-molding analysis and accurately map it onto the structural FEA models of both considered specimen configurations. The mapped FOT data were then used for local orientation averaging of the FOMM properties, enabling anisotropic stiffness to be incorporated into the FEA. The second goal was to evaluate the stiffness variation in SFRT with respect to the loading angle, which represents one of the key characteristics of anisotropic material behavior. This phenomenon was analyzed using an ISO 294-5 [36] compliant injection-molded plate, from which ISO 3167 [32] type 1B dog-bone specime ns were virtually extracted at different orientations relative to the flow direction. A graphical representation of this procedure is shown in Figure 9. The designed feed system of the injection-molded plate ensured smooth and uniform cavity filling. Because the plate had a constant thickness and no significant cross-sectional changes along the flow path, a region with a relatively uniform fiber orientation state was obtained. This enabled the extraction of virtual specimens at different angles relative to the flow direction while minimizing additional disturbances associated with local geometry variations.

**Figure 9:**
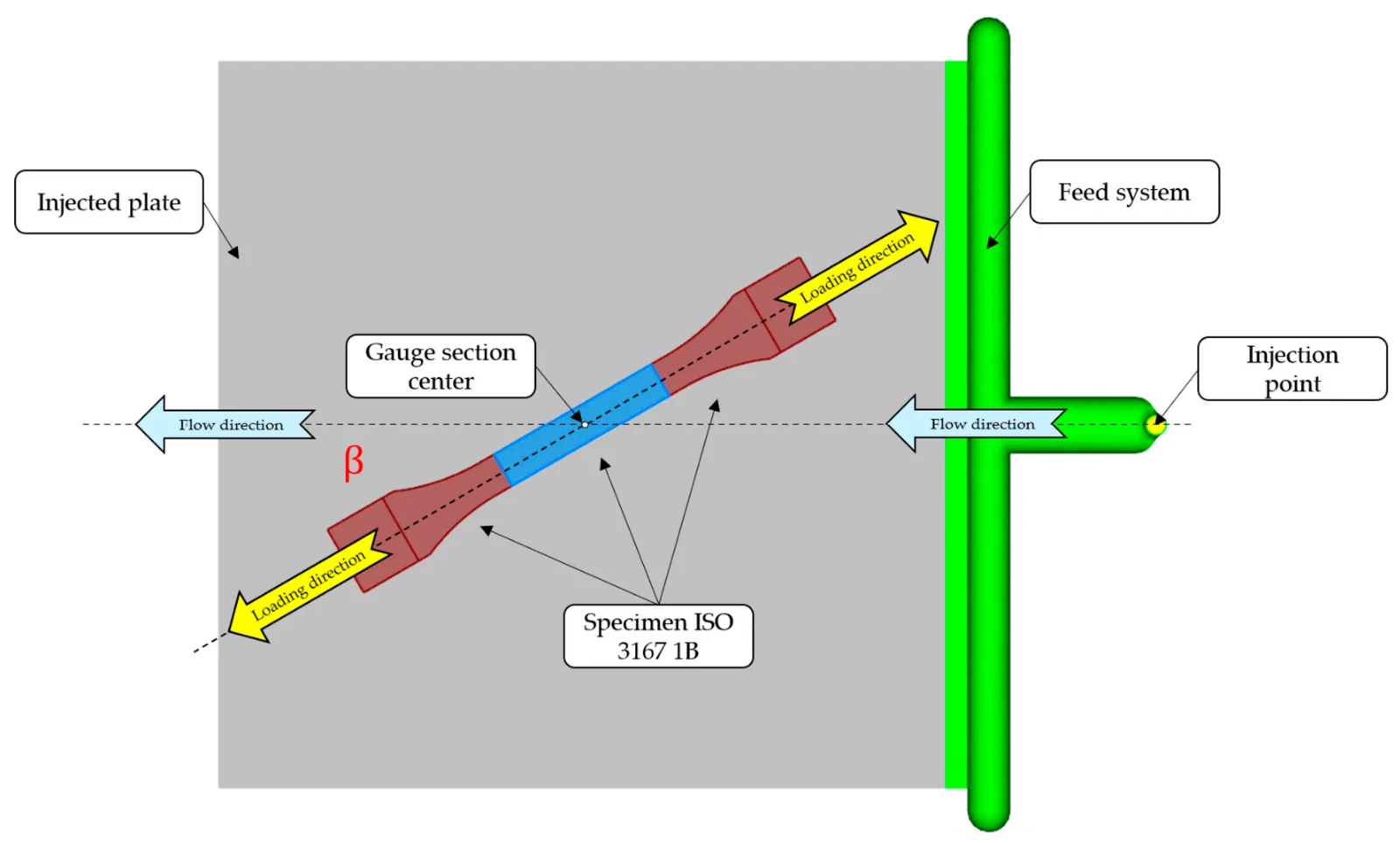
Numerical model used to simulate the production of an ISO 294-5 [36] compliant injection-molded plate, from which ISO 3167 [32] type 1B specimens were virtually extracted for tensile-test FEA.

#### 3.3.1. Injection-Molding Simulation Results

The injection-molding simulations provided the filling patterns shown in Figure 10 for the directly injection-molded specimen and in Figure 11 for the injection-molded plate. In both cases, the dominant melt flow direction was aligned with the longitudinal axis of the mold cavity. During the simulations, the result “shear stress at wall” was recorded. In the context of the filling analysis, this quantity represents the shear stress acting at the interface between the flowing melt and the frozen near-wall layer and is related to the local pressure gradient. According to [37], this result reflects one of the local flow-related factors influencing the development of molecular and fiber orientation in the melt adjacent to the frozen layer. However, the final fiber orientation state is determined by the combined effects of the local flow history, fountain-flow phenomena, fiber-fiber interactions, and rotary diffusion mechanisms. The distributions of the wall shear stress obtained for the two specimen configurations are presented in Figure 12 and Figure 13.

The final distributions of the first principal FOT value, *λ*_1_, for both specimen preparation routes are presented in Figure 14. The results show that the directly injection-molded specimen exhibits a higher degree of fiber orientation, especially in the gauge section and in the grip area located on the injection-gate side. This behavior is consistent with the dominant longitudnal flow pa3ttern observed during cavity filling. A more detailed discussion of the obtained FOT distributions is provided in the following subsection.

**Figure 10:**
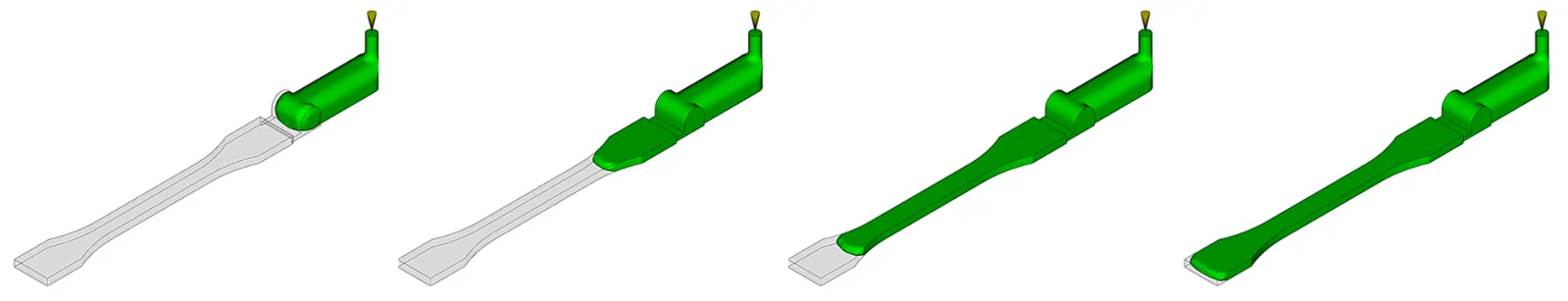
Filling stages of the molding simulation for a directly injection-molded ISO 3167 [32] type 1B dog-bone specimen with a single-sided gate.

**Figure 11:**
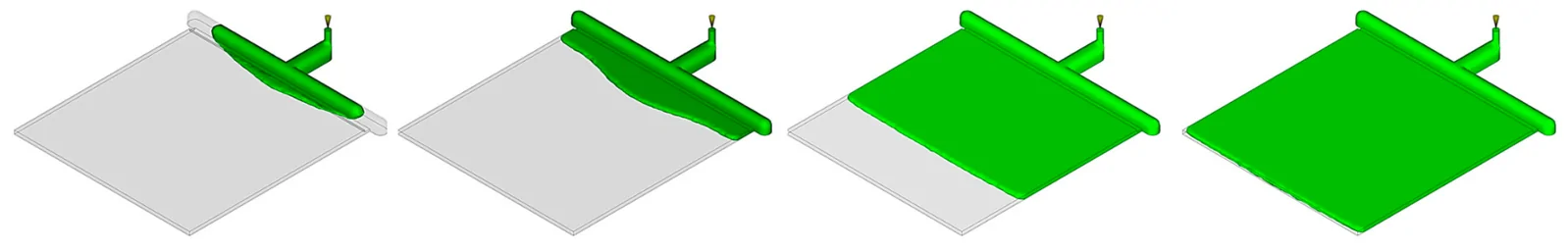
Selected stages of the molding simulation of an ISO 294-5 [36] compliant plate used for the virtual extraction of dog-bone specimens for tensile-test FEA.

**Figure 12:**
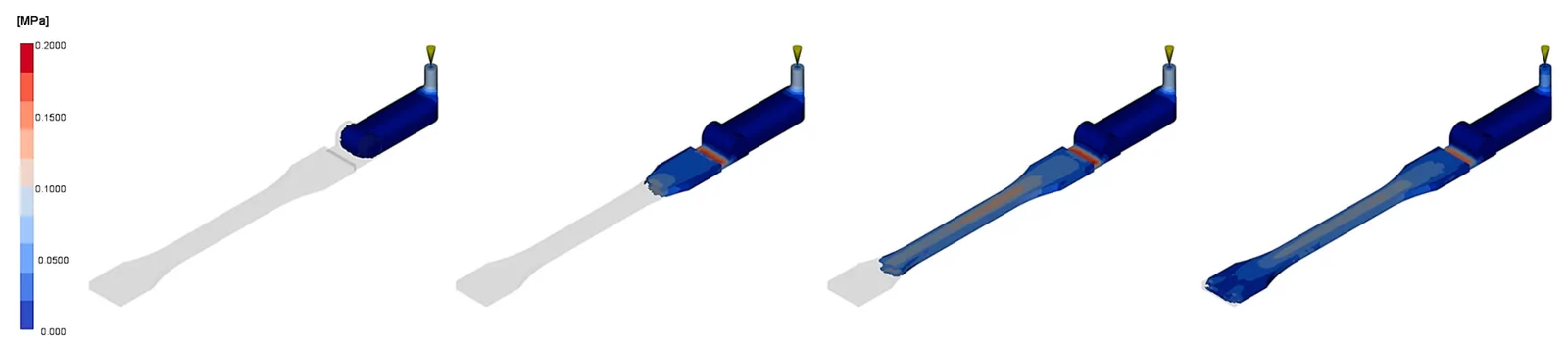
Shear stress at wall at different stages of cavity filling obtained from the molding simulation for a directly injection-molded ISO 3167 [32] type 1B dog-bone specimen with a single-sided gate.

**Figure 13:**
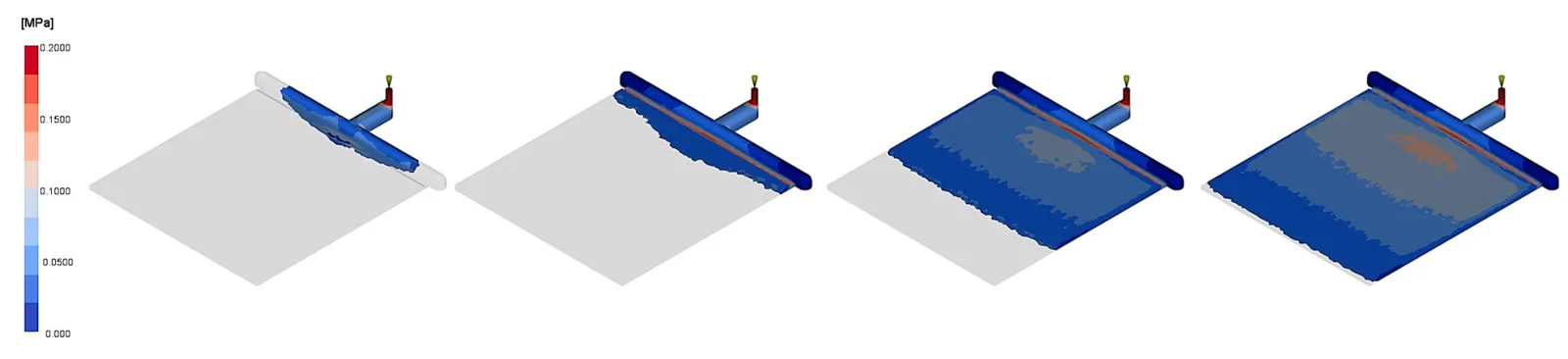
Shear stress at wall at different stages of cavity filling obtained from the molding simulation of an ISO 294-5 [36] compliant plate used for the virtual extraction of dog-bone specimens for tensile-test FEA.

**Figure 14:**
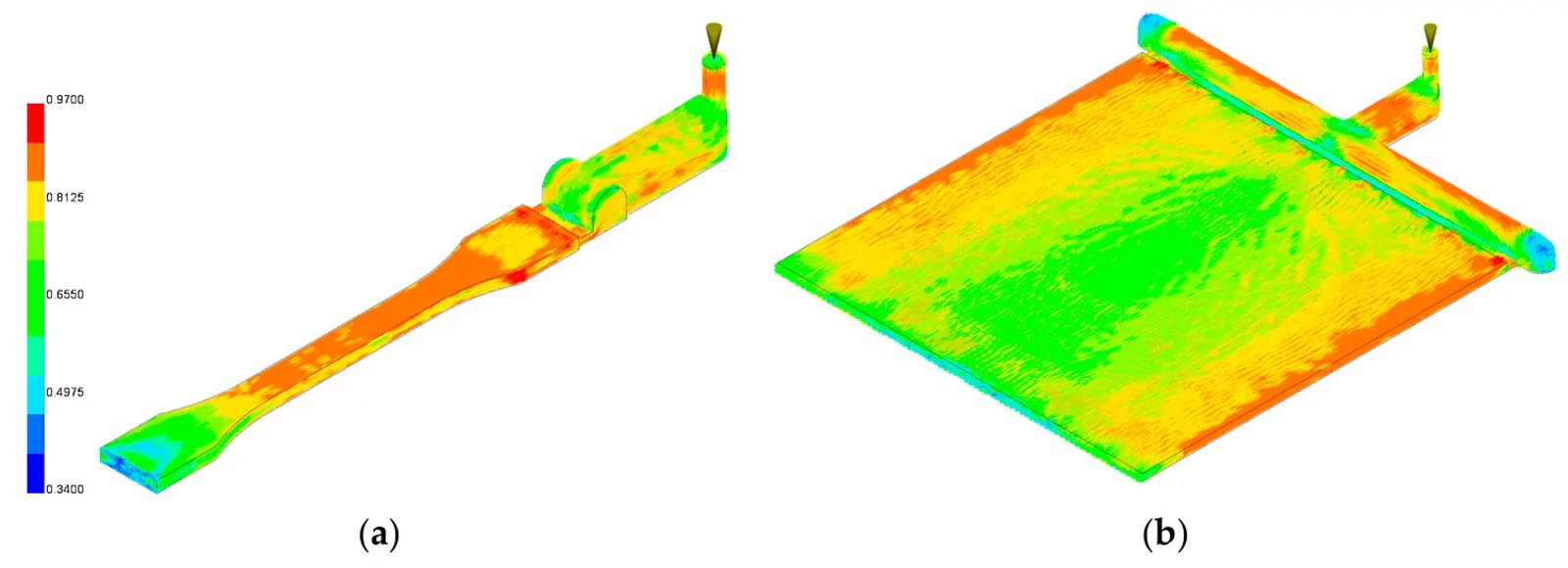
The 1st principal FOT value (*λ*_1_) obtained from injection-molding simulations for: (**a**) a directly injection-molded specimen with a single-sided gate; (**b**) an injection-molded plate.

#### 3.3.2. Effect of Specimen Preparation Route on the Fiber Orientation State

The spatial distribution of the FOT represents the principal manufacturing-induced information transferred from the injection-molding simulation to the structural FEA model. To initially demonstrate the influence of the specimen preparation route on the predicted fiber orientation state, a comparison was performed between a directly injection-molded dog-bone specimen and a specimen virtually machined from an injection-molded plate. In both cases, the specimen axis was aligned with the nominal flow direction, corresponding to *β* = 0° orientation (Figure 9). The resulting FOT distributions are presented in Figure 15.

The comparison of the first principal FOT value, *λ*_1_, obtained from the injection-molding analyses (Figure 14) with the corresponding values mapped onto the FEA models (Figure 15) revealed good agreement in both global and local distributions for the two considered specimen configurations. The observed agreement indicates that the developed in-house software is capable of transferring process-induced FOT data from the injection-molding simulation to the structural FEA model while preserving the main characteristics of the orientation distribution.

The directly injection-molded specimen, shown on the left-hand side of Figure 15, exhibits a stronger principal fiber orientation, as indicated by the larger values of *λ*_1_. The predominant alignment of the associated principal direction with the longitudinal specimen axis is confirmed by the coordinate-system-based FOT components discussed in Section 3.1. In addition, a nonuniform fiber orientation distribution can be observed in the grip regions. The grip located near the feed system (on the sample’s right side) shows a higher degree of orientation than the opposite grip. This effect can be attributed to the local reduction in cross-sectional area between the gate and the specimen cavity. As the melt enters the cavity through this restricted region, the local flow velocity and shear rate increase, which promotes stronger fiber alignment in the flow direction. Further downstream, the flow through the gauge section remains relatively smooth and uniform, resulting in a more homogeneous FOT distribution along the specimen length. This distribution is disturbed again near the rounded transition to the opposite grip, where the flow channel expands, the shear rate decreases, and the melt eventually reaches the end of the cavity. Consequently, the two grip regions exhibit different local fiber orientation states.

In contrast, the specimen virtually machined from the injection-molded plate shows a smoother and more uniform FOT distribution over its entire length. Compared with the directly injection-molded specimen, the machined specimen is characterized by lower *λ*_1_ values and higher *λ*_2_ values, indicating a less strongly concentrated principal orientation state. The coordinate-system-based components presented in Section 3.1 additionally show a lower *a*_11_ contribution and a higher *a*_22_ contribution, confirming weaker preferential alignment along the specimen axis and a more pronounced transverse in-plane orientation component. This difference results from the fact that the machined specimen is extracted from a larger plate region in which the flow is less affected by the local geometric restrictions characteristic of a directly injection-molded dog-bone cavity.

This comparison confirms that the specimen manufacturing route has a significant influence on the resulting fiber orientation state and, consequently, on the effective anisotropic thermomechanical properties. Therefore, precise knowledge of the SFRT specimen preparation process is essential when interpreting supplier-provided material data or in-house experimental results. From the perspective of structural FEA, this information is important because thermomechanical characteristics obtained from standardized tests represent the effective response of a specific process-dependent fiber orientation state, rather than a general material property independent of manufacturing conditions. If the corresponding FOT state is not known or is incorrectly assumed, the mapped anisotropic stiffness may not accurately represent the material behavior of the analyzed component.

#### 3.3.3. Orientation-Dependent SFRT Stiffness and Testing-Condition Effects in Structural FEA

Finally, following the scheme presented in Figure 9, the dependence of SFRT stiffness on the loading angle was evaluated to verify whether the developed mapping software can capture this effect in structural FEA.

In addition to the three temperature levels used for the detailed calibration and error assessment, the structural FEA demonstration included an intermediate temperature of 80 °C. The required composite-level reference properties at 80 °C were obtained from the same temperature-dependent datasets shown in Figure 2, Figure 3 and Figure 4, and the corresponding FOMM dataset was generated using the inverse-identification procedure described in Section 2.3. The 80 °C condition was included only in the structural demonstration to provide a more continuous representation of the temperature-dependent stiffness response.

The results presented in Figure 16 demonstrate the pronounced anisotropic response of PA66-GF30 resulting from the process-induced fiber orientation state. For both moisture conditions, the effective Young’s modulus decreased continuously as the loading angle increased from *β* = 0° to 90°. The highest stiffness was obtained at *β* = 0°, corresponding to loading along the nominal flow direction and the dominant average fiber orientation, whereas the lowest stiffness occurred at *β* = 90°, corresponding to transverse loading.

In the dry-as-molded state, the effective Young’s modulus decreased by approximately 35% to 85% when the loading direction changed from *β* = 0° to 90°, depending on the exposure temperature. A similar angular dependence was observed for the conditioned PA66-GF30, for which the reduction ranged from approximately 33% to 87%. In both moisture states, higher exposure temperatures resulted in a more pronounced stiffness reduction with increasing loading angle. These observations indicate that the stiffness of the investigated SFRT material is highly sensitive to the loading direction and that the anisotropic response is directly linked to the process-induced fiber orientation state.

As discussed above, the reduction in stiffness becomes more pronounced with increasing exposure temperature, which can be attributed to the softening of the PA66 matrix. In the conditioned state, this effect is further intensified by moisture absorption. Water absorbed by the polyamide matrix acts as a plasticizer, increases molecular mobility, and shifts the apparent mechanical transition temperature, *T*_a_, toward lower temperatures. For the studied SFRT, *T*_a_ was estimated to be approximately 45 °C for the dry-as-molded material and approximately 5 °C for the conditioned material. This phenomenon is clearly reflected in the stiffness trends obtained at 20 °C. For the dry-as-molded material, the exposure temperature remains below the estimated *T*_a_, and the stiffness response therefore remains relatively high. In contrast, for the conditioned material, 20 °C is above the estimated *T*_a_, which leads to a substantial decrease in stiffness. Therefore, the pronounced stiffness reduction observed for the conditioned material at room temperature should be interpreted as a consequence of the moisture-induced shift in the glass-transition-related softening range.

The angular stiffness-sensitivity measure was calculated for both moisture conditions as

(20) $$S_{E}\left(\beta \right)=-\frac{1}{E\left(0\right)}\frac{\mathrm{dE}\left(\beta \right)}{\mathrm{d\beta}}\cdot 100\%,$$

where *S_E_*(*β*) denotes the percentage loss of effective stiffness per one-degree increase in the loading angle. Positive values indicate a decrease in stiffness with increasing *β*. The resulting trends, presented in Figure 17, provide an additional measure of the orientation-dependent stiffness degradation and illustrate the combined effects of loading direction, exposure temperature, and moisture condition on the mechanical response of SFRT. The derivative was evaluated numerically from a spline interpolation of the discrete FEA-derived stiffness values.

For the analyzed PA66-GF30 material and the considered fiber-orientation state, the maximum sensitivity of effective stiffness to changes in loading direction occurred at *β* values of approximately 30° to 33°. Depending on temperature and moisture condition, the maximum value ranged approximately from 0.8 to 1.9% per degree. Below this range, the stiffness reduction per degree increased with *β*, indicating progressively greater sensitivity to deviations from the dominant fiber orientation. Beyond the peak-sensitivity range, the stiffness reduction per degree gradually decreased, although the effective stiffness continued to decline with increasing *β*.

A strongly concentrated fiber orientation state is beneficial primarily when the dominant loading direction is aligned with the eigenvector associated with the largest principal FOT value, *λ*_1_. The value *λ*_1_ quantifies the degree of orientation along that principal direction, whereas the corresponding eigenvector defines its spatial orientation.

If the orientation is evaluated in a specified coordinate system, the corresponding tensor components *a_ij_* should additionally be considered to determine the alignment relative to the local structural axes. Under off-axis loading, a highly oriented reinforcement state may result in a substantial reduction in effective stiffness relative to loading along the dominant fiber orientation. Therefore, the objective of process optimization should not be limited to maximizing fiber orientation, but should instead focus on matching the local fiber orientation state to the expected load paths.

This observation may contradict a simplified engineering interpretation in which a high degree of fiber orientation is automatically considered advantageous regardless of the local loading direction. In reality, strong fiber alignment reinforces one preferred direction while reducing the relative contribution of reinforcement in other directions. As a result, the mechanical benefit of fiber orientation must always be evaluated with respect to the local stress state and the expected service loading direction. Therefore, the present analysis indicates that maximizing fiber alignment alone should not be considered a universal optimization objective for injection-molded SFRT structures.

Finally, the FEA results provide an initial indication that the proposed approach can reproduce the expected orientation-dependent stiffness trends. They also support the feasibility of the developed numerical framework for incorporating process-induced local fiber orientation and environmental factors, such as temperature and moisture, into the prediction of thermomechanical properties. This capability provides the basis for subsequent local static and fatigue strength assessments of injection-molded SFRT components.

## 4. Conclusions

This study developed an integrated finite element framework for injection-molded short-fiber-reinforced thermoplastics that accounts for process-induced anisotropy, temperature, and moisture. The framework combines established micromechanical formulations, a fully oriented material model, process-derived fiber orientation tensors, and orientation averaging to generate spatially varying orthotropic thermomechanical properties for structural FEA. An inverse-identification route was additionally introduced for applications in which complete temperature- and moisture-dependent constituent properties are unavailable.

The analysis demonstrated that specimen manufacturing history is essential for interpreting composite-level material data. The directly injection-molded specimen exhibited a stronger longitudinal orientation than the specimen machined from an injection-molded plate. The plate also developed a layered through-thickness structure, with preferential orientation along the nominal flow direction in the outer regions and a transverse in-plane dominant orientation near the mid-plane. Supplier-provided and experimentally measured properties should therefore be interpreted as the effective response of a particular process-dependent orientation state rather than as manufacturing-independent material characteristics.

The accuracy of the non-calibrated FOMM depended strongly on property type and environmental condition. The longitudinal modulus was reproduced within the adopted ±10% engineering range at −20 °C and 20 °C, but was underpredicted by approximately 23% at 120 °C. The maximum transverse modulus deviation was 31.0%, while the maximum strength deviation was 51.4%. Particularly large thermal expansion discrepancies were also obtained at 120 °C. These results indicate that the constituent-based route becomes less reliable when strongly temperature- and moisture-dependent matrix behavior dominates the composite response.

Inverse identification substantially improved agreement with the selected composite-level targets. The effective longitudinal Young’s modulus and tensile strength were reproduced within ±0.4%. Their agreement represents calibration quality rather than independent prediction because both properties were included in the objective function. The non-targeted transverse modulus and strength remained within ±5.4% and ±1.9% of the auxiliary estimates, respectively, indicating internal directional consistency of the calibrated response.

For the dry-as-molded state, both directional thermal expansion coefficients remained within ±5.2% of their targets. For the conditioned state, the corresponding deviations remained within ±5.9%; however, these results describe agreement with targets adopted from the dry-as-molded dataset and do not constitute validation against independently measured conditioned-state properties. The equal and opposite residual deviations of the two directional coefficients reflect the compromise associated with using a single matrix thermal expansion coefficient to reproduce both responses.

The supplementary closure-sensitivity assessment showed that replacing OSC with OFC did not change the principal qualitative conclusions. Tensile strength and thermal expansion were comparatively insensitive to the closure selection, with maximum differences of 5.5% and 3.9%, respectively. The elastic moduli showed greater sensitivity, reaching 13.0% in the dry-as-molded state and 13.7% in the conditioned state for the transverse modulus of the specimen machined from the plate at 120 °C. Closure-related uncertainty should therefore be considered when quantitative stiffness predictions are required for less strongly aligned orientation states at elevated temperature.

The structural FEA demonstrations reproduced the expected dependence of stiffness on loading direction, temperature, and moisture condition. Stiffness was highest when the loading direction was aligned with the dominant fiber orientation and decreased under off-axis loading. Consequently, maximizing fiber alignment alone is not a sufficient process-optimization objective; the local orientation state should instead be matched to the expected structural load paths and service environment.

The results demonstrate successful calibration and internal consistency of the proposed framework, but independent validation of its full three-dimensional anisotropic response requires directional measurements not used during identification. The main remaining uncertainties concern the auxiliary transverse reference values, the assumed conditioned-state thermal expansion targets, the unknown manufacturing history of the thermal expansion specimens, and the adopted micromechanical and representative-FOT assumptions. Accordingly, the identified matrix properties should be regarded as effective parameters of the complete modeling framework rather than intrinsic properties of unreinforced PA66.

Despite these limitations, the developed approach provides a practical means of introducing process-induced local anisotropy and environmental effects into structural FEA when complete anisotropic material datasets are unavailable. Future work should include directional mechanical and thermal testing of specimens with experimentally characterized orientation states, extension to time-dependent constitutive behavior, multidirectional failure assessment, and incorporation of process-related effects such as weld lines, voids, and residual stresses.

## Figures and Tables

**Figure 1:**
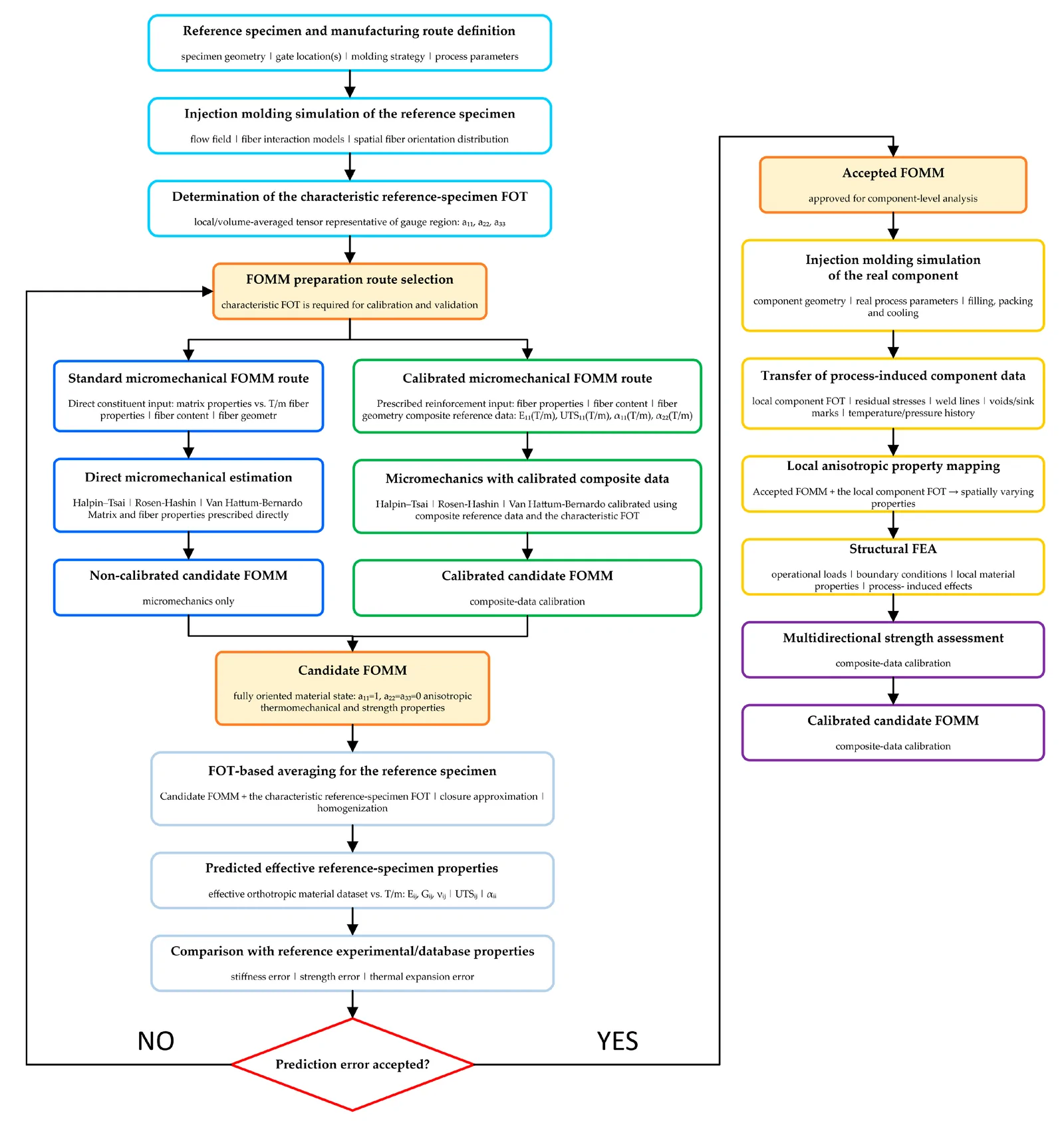
Advanced FEA workflow for SFRT components integrating process-induced FOT data, non-calibrated and calibration-based FOMM preparation, anisotropic property mapping, and multidirectional strength assessment. *T*/*m* denotes temperature- and moisture-dependent effects.

**Figure 2:**
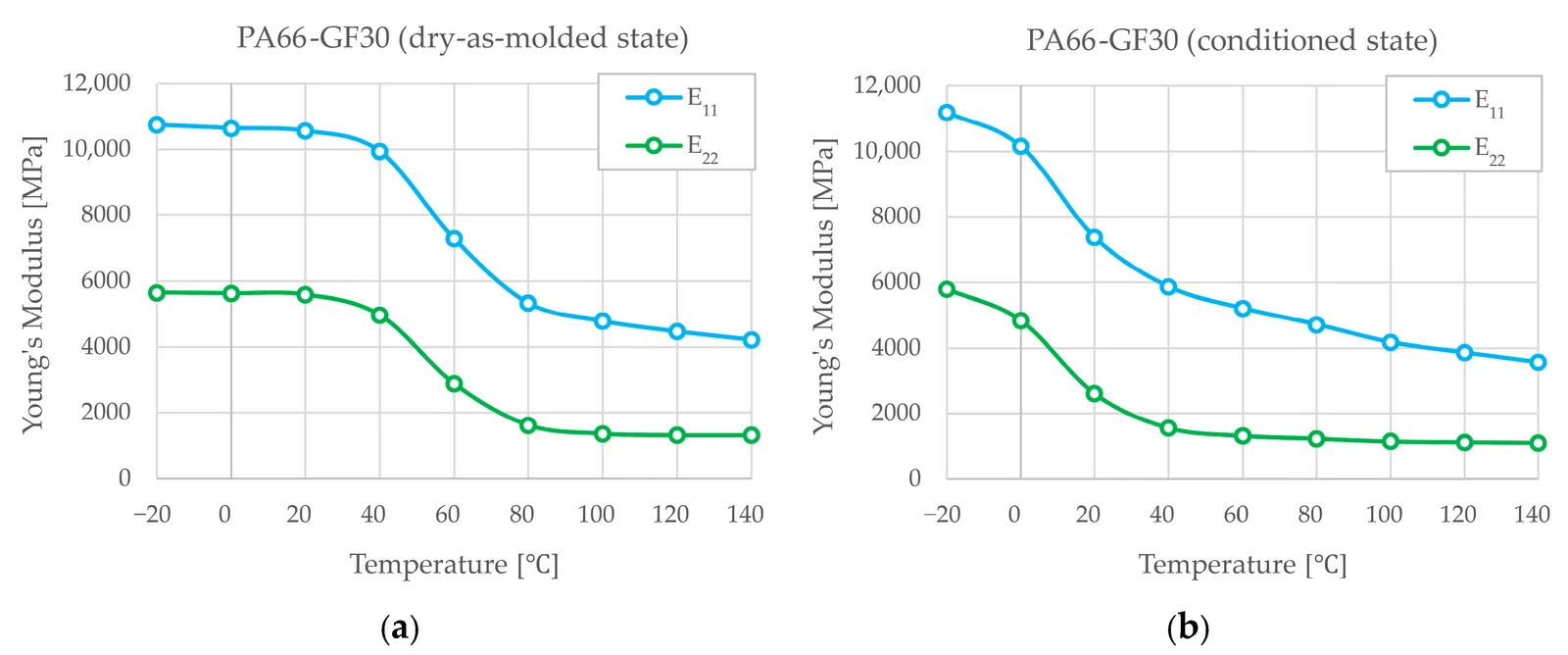
Young’s modulus of the investigated PA66-GF30 as a function of exposure temperature for two moisture conditions: (**a**) dry-as-molded state; (**b**) conditioned state. The longitudinal values, $E_{11}^{\mathrm{ref}}$, were derived from data available in the CAMPUS Plastics database [24], whereas the transverse values, $E_{22}^{\mathrm{ref},\mathrm{aux}}$, were obtained using the model-assisted auxiliary estimation procedure described in Section 2.2.1.

**Figure 3:**
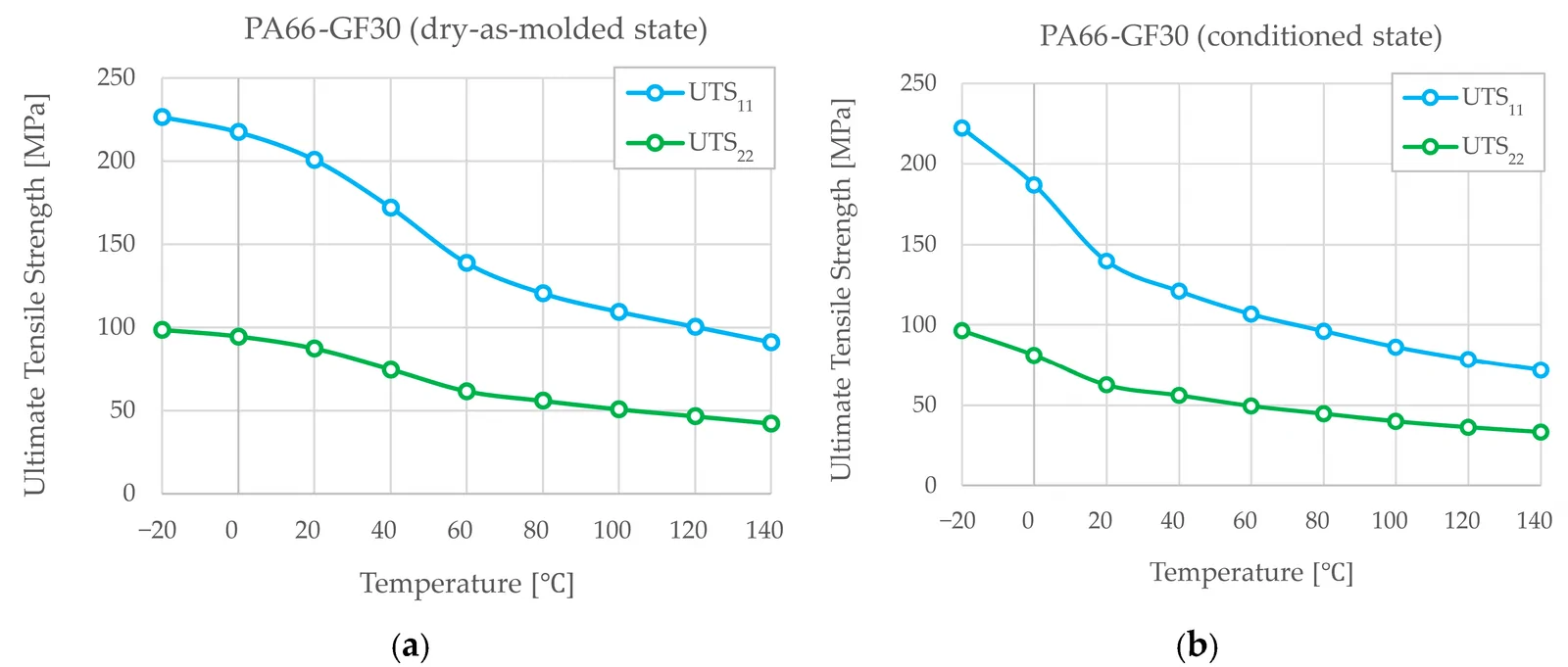
Ultimate tensile strength of the investigated PA66-GF30 as a function of exposure temperature for two moisture conditions: (**a**) dry-as-molded state; (**b**) conditioned state. The longitudinal values, $\mathrm{UT}S_{11}^{\mathrm{ref}}$, were derived from data available in the CAMPUS Plastics database [24], whereas the transverse values, $\mathrm{UT}S_{22}^{\mathrm{ref},\mathrm{aux}}$, were obtained using the model-assisted auxiliary estimation procedure described in Section 2.2.1.

**Figure 4:**
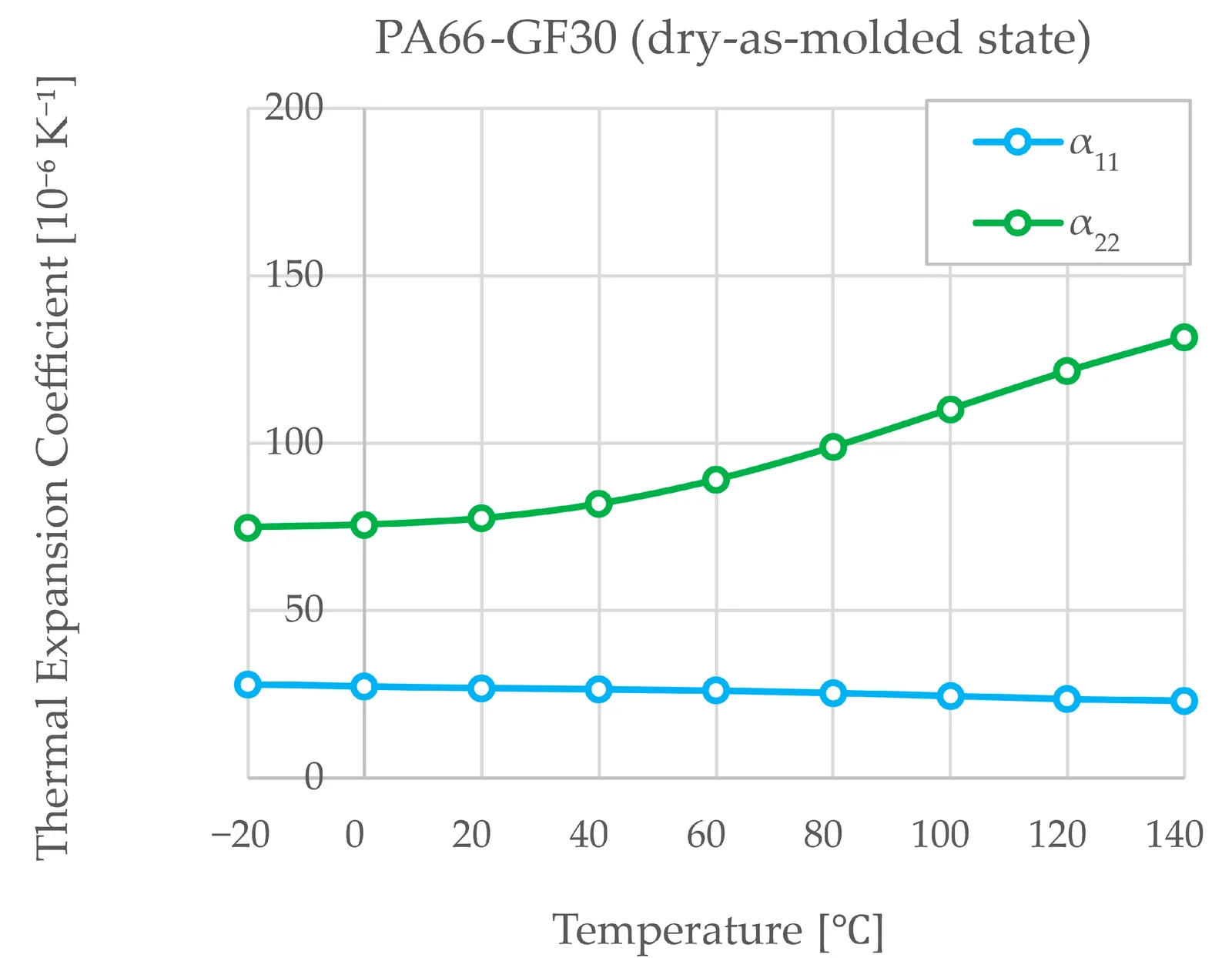
Temperature-dependent coefficient of linear thermal expansion of PA66-GF30 under dry-as-molded conditions, based on CAMPUS Plastics data [24].

**Figure 5:**
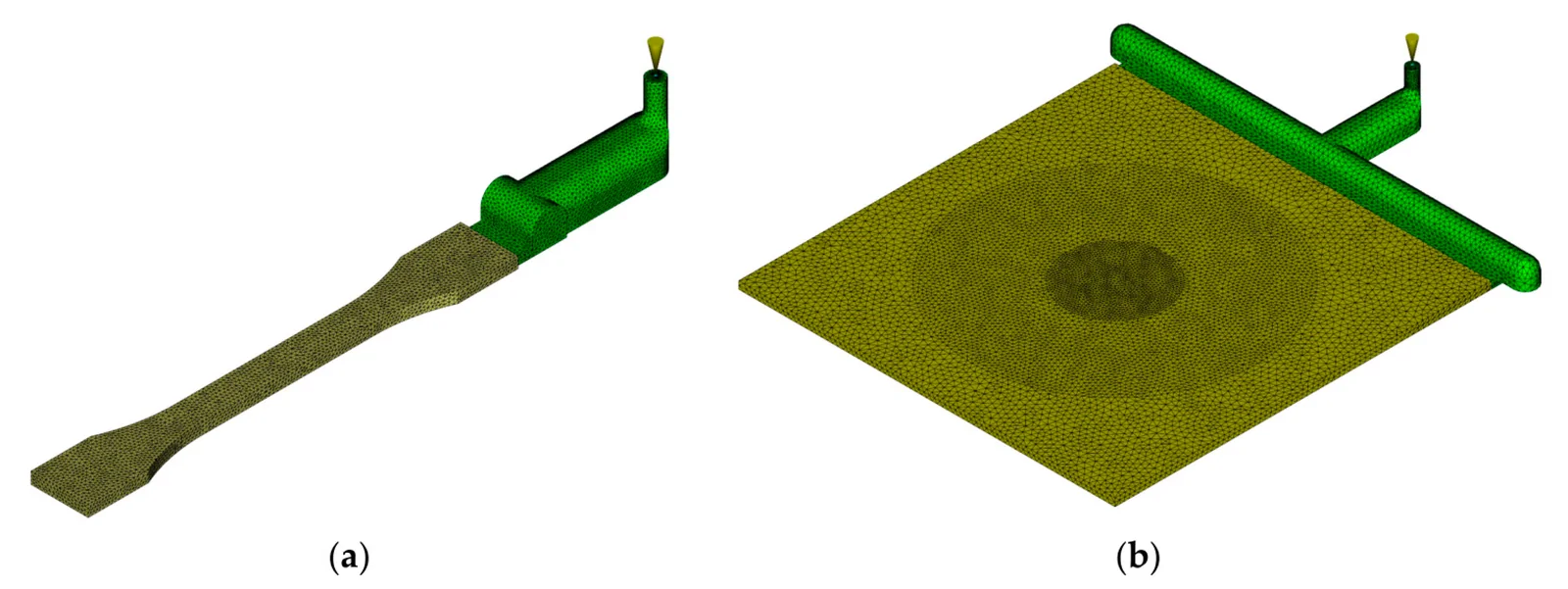
Simulation models of two common SFRT specimen preparation methods: (**a**) a directly injection-molded specimen with a single-sided gate; (**b**) an injection-molded plate.

**Figure 6:**
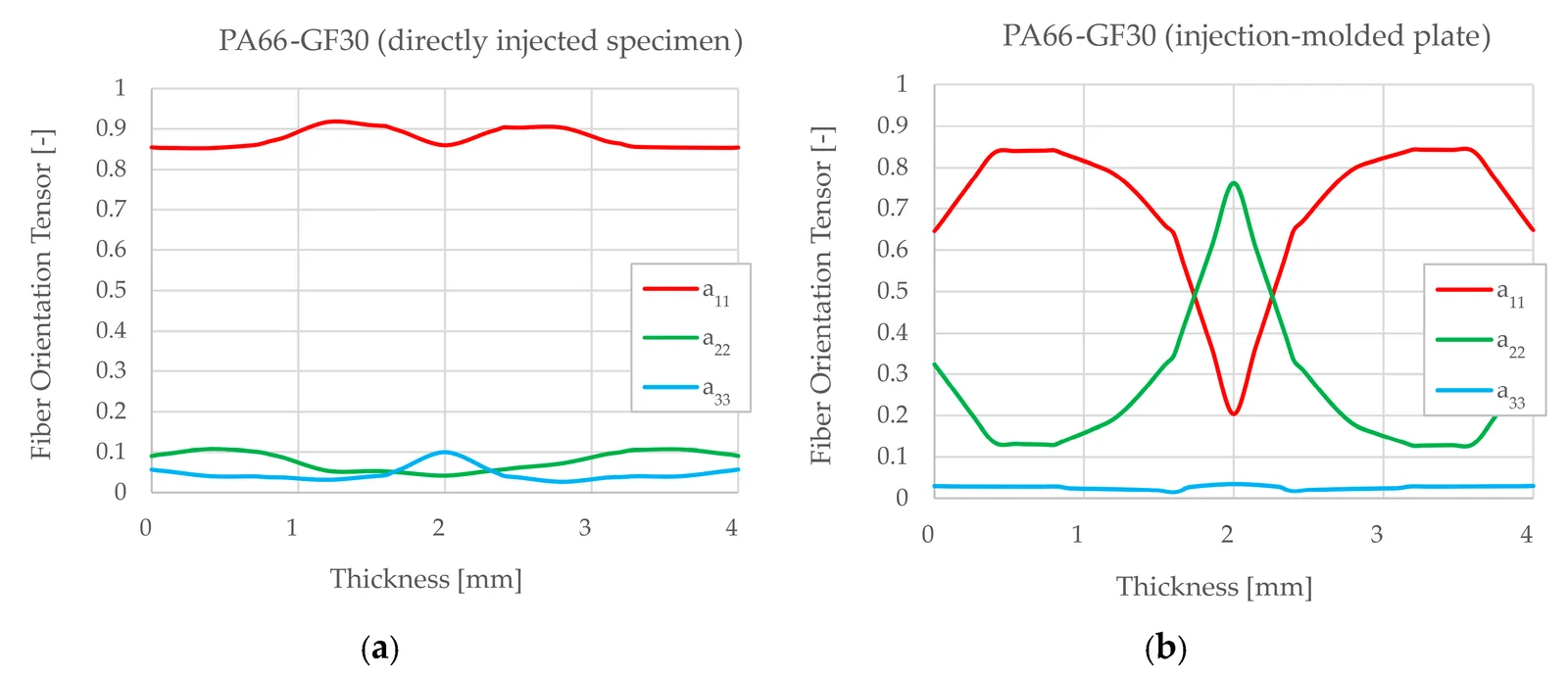
Fiber orientation tensor components (at the center of the specimen gauge section) obtained from injection-molding simulations for: (**a**) a directly injection-molded specimen with a single-sided gate; (**b**) an injection-molded plate.

**Figure 7:**
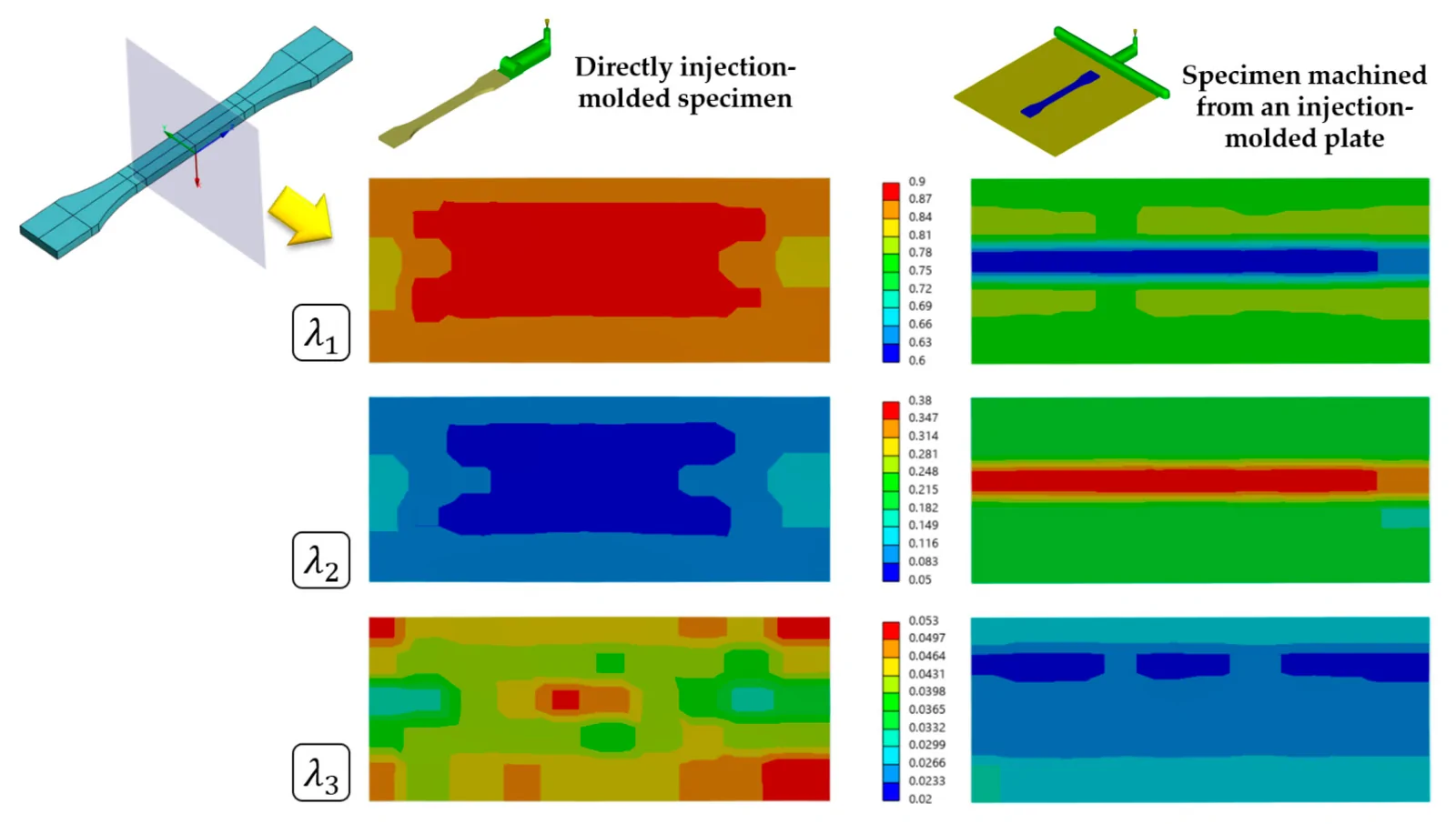
Principal values (*λ*_1_ ≥ *λ*_2_ ≥ *λ*_3_) of the FOT mapped onto the finite elements of the structural FEA models for the directly injection-molded specimen and the specimen machined from the injection-molded plate. The displayed cross-sections are located at the longitudinal center of the gauge section. The presented values are averaged over each finite element.

**Figure 8:**
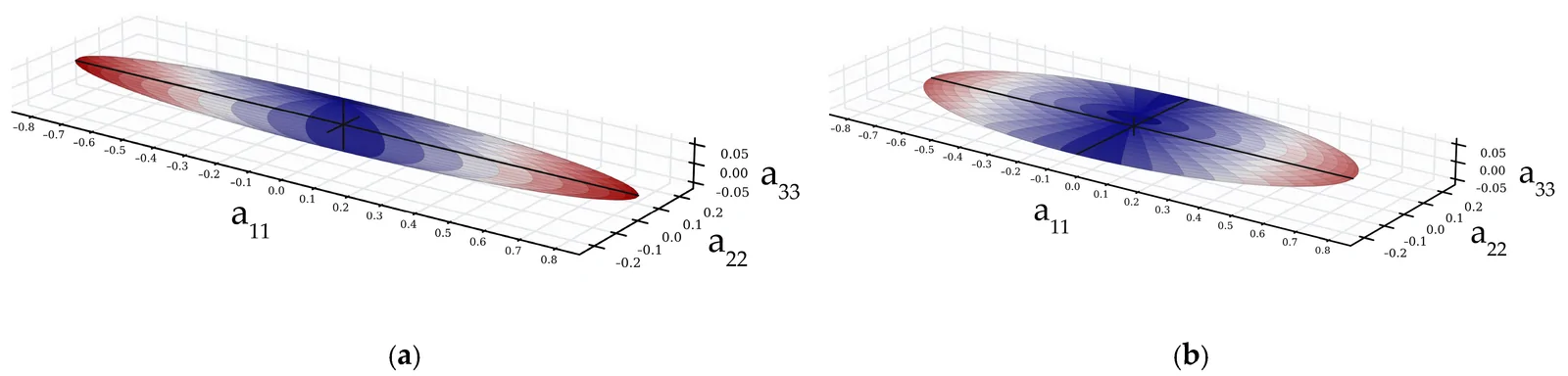
Direct representation of the volume-averaged diagonal fiber orientation tensor components *a*_11_, *a*_22_, and *a*_33_, expressed in the specimen-based coordinate system, over the gauge section for: (**a**) a directly injection-molded specimen with a single-sided gate; (**b**) a specimen machined from an injection-molded plate.

**Figure 15:**
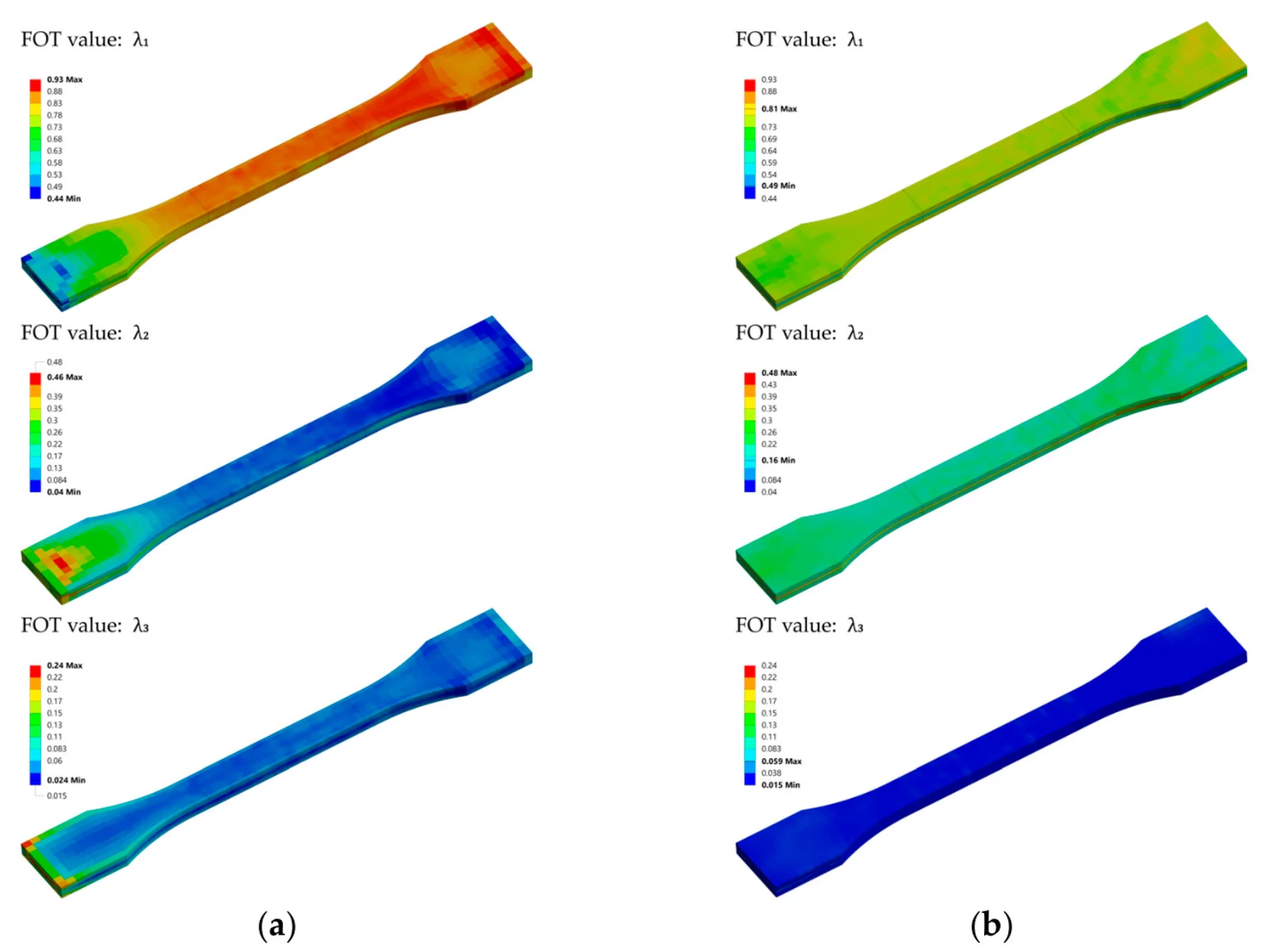
Comparison of the principal FOT values mapped onto the FEA models for specimens oriented along the nominal flow direction: (**a**) directly injection-molded dog-bone specimen; (**b**) specimen virtually extracted from an ISO 294 [36] injection-molded plate at *β* = 0°.

**Figure 16:**
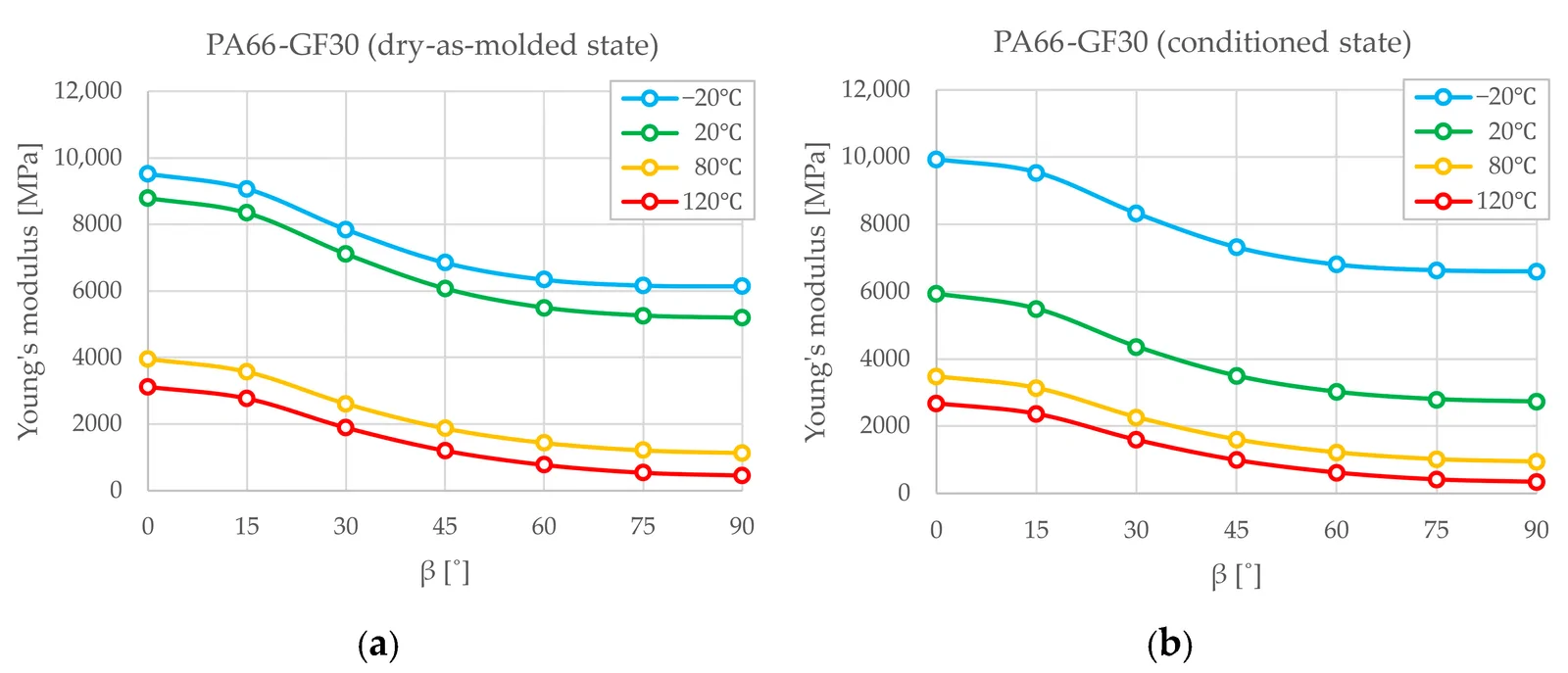
FEA-derived effective Young’s modulus of ISO 3167 [32] type 1B dog-bone specimens as a function of the loading angle relative to the nominal flow direction, *β*, and exposure temperature for two moisture conditions: (**a**) dry-as-molded; (**b**) conditioned.

**Figure 17:**
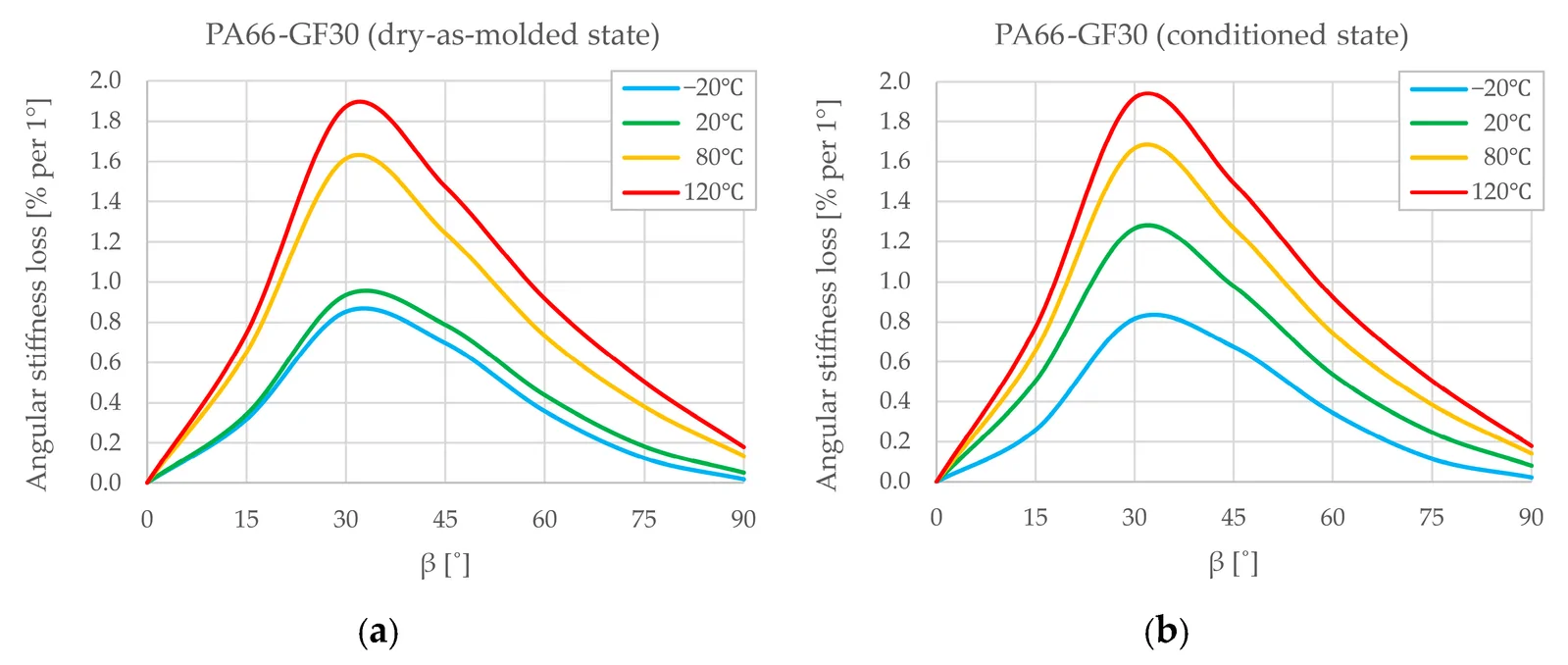
Normalized angular loss of effective stiffness, *S_E_*(*β*), derived from the FEA results as a function of the loading angle relative to the nominal flow direction for different exposure temperatures and two moisture conditions: (**a**) dry-as-molded; (**b**) conditioned.

**Table 1:** Non-calibrated micromechanics-based FOMM properties of PA66-GF30 obtained using the initial constituent properties, assuming complete fiber alignment along the local *x*_1_ axis and transverse isotropy in the *x*_2_–*x*_3_ plane.

			DRY-AS-MOLDED State			CONDITIONED State		
Temperature			−20 °C	20 °C	120 °C	−20 °C	20 °C	120 °C
Elastic constants	$E_{11}^{\mathrm{FOMM}}$	MPa	11,514	11,292	4002	11,968	8151	3415
	$E_{22}^{\mathrm{FOMM}}$	MPa	4714	4629	824	5126	2403	668
	$\nu_{12}^{\mathrm{FOMM}}$	-	0.36	0.36	0.44	0.39	0.43	0.45
	$\nu_{23}^{\mathrm{FOMM}}$	-	0.54	0.54	0.67	0.58	0.64	0.68
	$G_{12}^{\mathrm{FOMM}}$	MPa	1528	1501	247	1627	731	198
	$G_{23}^{\mathrm{FOMM}}$	MPa	1528	1501	247	1627	731	198
Strength constants	${\mathrm{UTS}}_{11}^{\mathrm{FOMM}}$	MPa	329	255	108	334	229	107
	${\mathrm{UTS}}_{22}^{\mathrm{FOMM}}$	MPa	116	86	44	116	86	44
Thermal expansion coefficients	$\alpha_{11}^{\mathrm{FOMM}}$	10^−6^ K^−1^	25	25	49	27	27	81
	$\alpha_{22}^{\mathrm{FOMM}}$	10^−6^ K^−1^	83	85	141	84	89	127

**Table 2:** Summary of reference thermomechanical properties for PA66-GF30. Own work based on data from the CAMPUS Plastics database [24]. The subscripts 11 and 22 refer to properties resolved along the specimen-based coordinate axes *x*_1_ and *x*_2_, respectively. The *x*_1_ axis is aligned with the longitudinal specimen axis, nominal mold-filling direction, and tensile loading direction of the reference specimen, whereas *x*_2_ is the transverse in-plane direction. The *x*_3_ axis corresponds to the through-thickness direction. These specimen directions differ from the local FOMM symmetry axes, except in the fully aligned configuration.

			DRY-AS-MOLDED State			CONDITIONED State		
Temperature			−20 °C	20 °C	120 °C	−20 °C	20 °C	120 °C
Elastic constants	$E_{11}^{\mathrm{ref}}$	MPa	10,745	10,563	4476	11,186	7380	3861
	$E_{22}^{\mathrm{ref},\mathrm{aux}}$	MPa	5653	5591	1317	5791	2608	1129
Strength constants	${\mathrm{UTS}}_{11}^{\mathrm{ref}}$	MPa	226	201	100	223	140	78
	${\mathrm{UTS}}_{22}^{\mathrm{ref},\mathrm{aux}}$	MPa	98	87	47	97	63	36
Thermal expansion coefficients	$\alpha_{11}^{\mathrm{ref}}$	10^−6^ K^−1^	28	27	24	28 **	27^**^	24 **
	$\alpha_{22}^{\mathrm{ref}}$	10^−6^ K^−1^	75	78	122	75 **	78^**^	122 **

*aux*: Reference value obtained using the auxiliary estimation procedure and used only to evaluate the non-targeted transverse mechanical predictions. ** Assumed to be equal to the corresponding dry-as-molded reference value at the same temperature and used as a conditioned-state calibration target.

**Table 3:** Experimental domain of specimens machined from injection-molded plates and used to develop the auxiliary transverse-property estimation procedure.

Matrix Family	Nominal Reinforcement	Test-Temperature Range	Moisture Condition
PBT	30 wt.% short glass fibers	−40 to 120 °C	-
PA6	35 wt.% short glass fibers	−40 to 120 °C	Dry
PA612	30 wt.% short glass fibers	23 °C	Dry and conditioned
PA6T/6I	50 wt.% short glass fibers	23 °C and 80 °C	Dry and conditioned
PPS	40 wt.% short glass fibers	−40 to 200 °C	-

**Table 4:** Minimum input data for FOMM preparation and component-level application.

Input Data		Non-Calibrated Route	Calibration-Based Route
Matrix Young’s modulus	*E_m_*(*T*,*h*)	Required	Initial value and bounds
Matrix Poisson’s ratio	*ν_m_*(*T*,*h*)	Required	Initial value and bounds
Matrix tensile strength	*UTS_m_*(*T*,*h*)	Required for strength analysis	Initial value and bounds
Matrix thermal expansion coeff.	*α_m_*(*T*,*h*)	Required for thermal analysis	Initial value and bounds
Fiber properties	*E_f_*, *ν_f_*, *UTS_f_*, *α_f_*	Required ^1^	Required ^1^
Fiber volume fraction	*V_f_*	Required	Required
Fiber aspect ratio	*a_r_* = *L_f_*/*d_f_*	Required	Required
Composite longitudinal modulus	$E_{11}^{\mathrm{ref}}(T,h)$	Not required	Required
Composite longitudinal strength	$\mathrm{UT}S_{11}^{\mathrm{ref}}(T,h)$	Not required	Required for strength calibration
Composite thermal expansion	$\alpha_{11}^{\mathrm{ref}}(T,h)$, $\alpha_{22}^{\mathrm{ref}}(T,h)$	Not required	Required for thermal calibration
FOT of the reference specimen	**A** *^rep^*	Not required ^2^	Required
Local component FOT field	**A**(**x**)	Required for component analysis	Required for component analysis

^1^ Only the fiber properties corresponding to the intended analysis scope are required. ^2^ Required only when the non-calibrated model is compared with specimen-level reference data. *T* and *h* denote temperature and moisture condition, respectively. If *V_f_* is unavailable, it can be calculated from the fiber mass fraction using the matrix and fiber densities.

**Table 5:** Volume-averaged diagonal components of the second-order fiber orientation tensor, expressed in the specimen-based coordinate system, for the specimen gauge section.

2nd Order Orientation Tensor	Directly Injection-Molded	Machined from Plate
*a*_11_	0.866	0.707
*a*_22_	0.069	0.267
*a*_33_	0.065	0.026

**Table 6:** Relative deviations (%) of the effective thermomechanical properties of PA66-GF30 after FOT-based orientation averaging using the non-calibrated FOMM.

			DRY-AS-MOLDED State			CONDITIONED State		
Temperature			−20 °C	20 °C	120 °C	−20 °C	20 °C	120 °C
Elastic constants	$\varepsilon_{E_{11}}$	%	−4.0%	−4.3%	−22.7%	−3.9%	−3.1%	−23.6%
	$\varepsilon_{E_{22}}^{\mathrm{aux}}$	%	−12.6%	−13.2%	−27.8%	−7.5%	0.5%	−31.0%
Strength constants	$\varepsilon_{{\mathrm{UTS}}_{11}}$	%	22.8%	5.8%	1.4%	25.4%	47.4%	28.4%
	$\varepsilon_{{\mathrm{UTS}}_{22}}^{\mathrm{aux}}$	%	26.1%	6.1%	7.4%	28.2%	51.4%	38.1%
Thermal expansion coefficients	$\varepsilon_{\alpha_{11}}$	%	−5.9%	0.1%	84.6%	1.1% **	−2.2% **	192.8% **
	$\varepsilon_{\alpha_{22}}$	%	8.8%	7.7%	18.2%	11.2% **	15.6% **	11.7% **

*aux*: Calculated relative to the reference value obtained using the auxiliary estimation procedure. ** Calculated relative to the conditioned-state thermal expansion target assumed to be equal to the corresponding dry-as-molded reference value at the same temperature.

**Table 7:** FOMM properties obtained using the identified matrix properties, assuming complete fiber alignment in the *x*_1_ direction and transverse isotropy in the *x*_2_–*x*_3_ plane.

			DRY-AS-MOLDED State			CONDITIONED State		
Temperature			−20 °C	20 °C	120 °C	−20 °C	20 °C	120 °C
Elastic constants	$E_{11}^{\mathrm{FOMM}}$	MPa	11,962	11,787	5156	12,411	8366	4459
	$E_{22}^{\mathrm{FOMM}}$	MPa	5120	5077	1182	5549	2523	957
	$\nu_{12}^{\mathrm{FOMM}}$	-	0.37	0.36	0.41	0.39	0.43	0.42
	$\nu_{23}^{\mathrm{FOMM}}$	-	0.55	0.53	0.63	0.57	0.65	0.64
	$G_{12}^{\mathrm{FOMM}}$	MPa	1653	1658	363	1762	764	291
	$G_{23}^{\mathrm{FOMM}}$	MPa	1653	1658	363	1762	764	291
Strength constants	${\mathrm{UTS}}_{11}^{\mathrm{FOMM}}$	MPa	276	247	108	275	159	84
	${\mathrm{UTS}}_{22}^{\mathrm{FOMM}}$	MPa	91	81	42	89	56	33
Thermal expansion coefficients	$\alpha_{11}^{\mathrm{FOMM}}$	10^−6^ K^−1^	25	25	26	26	28	26
	$\alpha_{22}^{\mathrm{FOMM}}$	10^−6^ K^−1^	80	80	115	78	79	114

**Table 8:** Relative deviations of the effective thermomechanical properties obtained after FOT-based orientation averaging of the FOMM generated using the identified matrix properties.

			DRY-AS-MOLDED State			CONDITIONED State		
Temperature			−20 °C	20 °C	120 °C	−20 °C	20 °C	120 °C
Elastic constants	$\varepsilon_{E_{11}}$	%	0.0%	0.3%	0.0%	0.0%	−0.4%	0.0%
	$\varepsilon_{E_{22}}^{\mathrm{aux}}$	%	−5.4%	−5.2%	1.5%	−0.3%	5.3%	−3.1%
Strength constants	$\varepsilon_{{\mathrm{UTS}}_{11}}$	%	0.0%	0.3%	0.0%	0.0%	0.4%	0.0%
	$\varepsilon_{{\mathrm{UTS}}_{22}}^{\mathrm{aux}}$	%	−0.6%	−1.0%	1.9%	−1.5%	−1.9%	1.6%
Thermal expansion coefficients	$\varepsilon_{\alpha_{11}}$	%	−5.1%	−1.2%	5.2%	−2.3% **	−2.0%^**^	5.9% **
	$\varepsilon_{\alpha_{22}}$	%	5.1%	1.2%	−5.2%	2.3% **	2.0%^**^	−5.9% **

*aux*: Calculated relative to the reference value obtained using the auxiliary estimation procedure. ** Calculated relative to the conditioned-state calibration target assumed to be equal to the corresponding dry-as-molded reference value at the same temperature.

**Table 9:** Symmetric absolute percentage differences, $\Delta_{X}^{\mathrm{cl}}$, between the effective thermomechanical properties obtained using the Orthotropic Smooth Closure approximation adopted in the primary analysis and the Orthotropic Fitted Closure approximation used in the supplementary sensitivity assessment for the dry-as-molded state.

DRY-AS-MOLDED State			Directly Injection-Molded			Machined from Plate		
Temperature			−20 °C	20 °C	120 °C	−20 °C	20 °C	120 °C
Elastic constants	$\Delta_{E_{11}}^{\mathrm{cl}}$	%	5.7%	5.6%	8.1%	6.7%	6.6%	10.0%
	$\Delta_{E_{22}}^{\mathrm{cl}}$	%	3.4%	3.3%	6.9%	7.3%	7.1%	13.0%
Strength constants	$\Delta_{{\mathrm{UTS}}_{11}}^{\mathrm{cl}}$	%	3.3%	3.3%	2.5%	3.4%	3.4%	3.0%
	$\Delta_{{\mathrm{UTS}}_{22}}^{\mathrm{cl}}$	%	1.5%	1.5%	2.9%	3.5%	3.4%	5.3%
Thermal expansion coefficients	$\Delta_{\alpha_{11}}^{\mathrm{cl}}$	%	0.7%	0.6%	3.1%	1.9%	1.9%	1.8%
	$\Delta_{\alpha_{22}}^{\mathrm{cl}}$	%	0.4%	0.4%	1.2%	1.8%	1.8%	3.8%

The calibrated FOMM datasets and the second-order FOTs were kept unchanged. No recalibration was performed using OFC.

**Table 10:** Symmetric absolute percentage differences, $\Delta_{X}^{\mathrm{cl}}$, between the effective thermomechanical properties obtained using the Orthotropic Smooth Closure approximation adopted in the primary analysis and the Orthotropic Fitted Closure approximation used in the supplementary sensitivity assessment for the conditioned state.

CONDITIONED State			Directly Injection-Molded			Machined from Plate		
Temperature			−20 °C	20 °C	120 °C	−20 °C	20 °C	120 °C
Elastic constants	$\Delta_{E_{11}}^{\mathrm{cl}}$	%	5.7%	7.5%	8.3%	6.6%	9.1%	10.4%
	$\Delta_{E_{22}}^{\mathrm{cl}}$	%	3.3%	5.3%	7.3%	7.0%	10.8%	13.7%
Strength constants	$\Delta_{{\mathrm{UTS}}_{11}}^{\mathrm{cl}}$	%	3.4%	3.0%	2.5%	3.4%	3.4%	3.0%
	$\Delta_{{\mathrm{UTS}}_{22}}^{\mathrm{cl}}$	%	1.5%	2.3%	3.1%	3.4%	4.7%	5.5%
Thermal expansion coefficients	$\Delta_{\alpha_{11}}^{\mathrm{cl}}$	%	0.6%	1.8%	3.4%	1.8%	0.9%	1.4%
	$\Delta_{\alpha_{22}}^{\mathrm{cl}}$	%	0.4%	0.8%	1.3%	1.7%	2.9%	3.9%

The calibrated FOMM datasets and the second-order FOTs were kept unchanged. No recalibration was performed using OFC.

## Data Availability

The data presented in this study are not publicly available due to confidentiality restrictions related to industrial data.

## Abbreviations

The following abbreviations are used in this manuscript:

APDL	ANSYS Parametric Design Language
DSC	Differential Scanning Calorimetry
DMA	Dynamic Mechanical Analysis
FEA	Finite Element Analysis
FOMM	Fully Oriented Material Model
FOT	Fiber Orientation Tensor
GF	Glass Fibers
OSC	Orthotropic Smooth Closure
OFC	Orthotropic Fitted Closure
PA66	Polyamide 66
PDF	Probability Density Function
SFRT(s)	Short-Fiber-Reinforced Thermoplastic(s)
*UTS*	Ultimate Tensile Strength
*UTS_m_*	Matrix ultimate tensile strength
α	Coefficient of Linear Thermal Expansion
*α_m_*	Matrix coefficient of linear thermal expansion
*β*	Loading Angle Relative to the Nominal Flow Direction
*E*	Young’s Modulus
*E_m_*	Matrix Young’s modulus
*ν_m_*	Matrix Poisson’s ratio
*T_a_*	Apparent Mechanical Transition Temperature
*T_g_*	Glass Transition Temperature
*λ_i_*	Ordered principal value of the second-order fiber orientation tensor
*a_ij_*	Component of the second-order fiber orientation tensor in the selected coordinate system
$\Delta_{X}^{\mathrm{cl}}$	Symmetric absolute percentage difference between a property obtained using OSC and the corresponding property recalculated using OFC
**p***_T_*_,_*_h_*	Matrix-property optimization vector containing *E_m_*, *ν_m_*, *UTS_m_*, and *α_m_* at temperature *T* and moisture condition *h*
**A***^rep^*	Representative fiber orientation tensor of the directly injection-molded specimen used for mechanical and thermal orientation averaging
**A**(**x**)	Spatially varying second-order fiber orientation tensor of the analyzed component
*J_T_*_,_*_h_*	Objective function at temperature *T* and moisture condition *h*
*r_k_*_,_*_T_*_,_*_h_*	Dimensionless relative calibration error for property *k* at temperature *T* and moisture condition *h*
Ω*_T_*_,_*_h_*	Admissible parameter domain at temperature *T* and moisture condition *h*
$X_{\mathrm{ij}}^{\mathrm{FOMM}}$	Directional property of the fully oriented material model
$X_{\mathrm{ij}}^{\mathrm{ref}}$	Reference property of the process-dependent specimen
$X_{\mathrm{ij}}^{\mathrm{ref},\mathrm{aux}}$	Auxiliary estimated reference property
${\hat{X}}_{\mathrm{ij}}^{\mathrm{eff}}$	Predicted effective property obtained by FOT-based orientation averaging
$\varepsilon_{X_{\mathrm{ij}}}$	Signed relative deviation from the specified reference value
$\varepsilon_{X_{\mathrm{ij}}}^{\mathrm{aux}}$	Signed relative deviation from an auxiliary estimated reference value
*S_E_*(*β*)	Angular stiffness-sensitivity measure
